# Toluene Dioxygenase-Catalyzed *cis*-Dihydroxylation of Quinolines: A Molecular Docking Study and Chemoenzymatic Synthesis of Quinoline Arene Oxides

**DOI:** 10.3389/fbioe.2020.619175

**Published:** 2021-02-12

**Authors:** Derek R. Boyd, Narain D. Sharma, Pui L. Loke, Jonathan G. Carroll, Paul J. Stevenson, Patrick Hoering, Christopher C. R. Allen

**Affiliations:** ^1^School of Chemistry and Chemical Engineering, Queen's University of Belfast, Belfast, United Kingdom; ^2^School of Biological Sciences, Queen's University of Belfast, Belfast, United Kingdom

**Keywords:** arene oxides, *cis*-dihydrodiols, dioxygenase, docking, biocatalysis

## Abstract

Molecular docking studies of quinoline and 2-chloroquinoline substrates at the active site of toluene dioxygenase (TDO), were conducted using Autodock Vina, to identify novel edge-to-face interactions and to rationalize the observed stereoselective *cis*-dihydroxylation of carbocyclic rings and formation of isolable *cis*-dihydrodiol metabolites. These *in silico* docking results of quinoline and pyridine substrates, with TDO, also provided support for the postulated *cis*-dihydroxylation of electron-deficient pyridyl rings, to give transient *cis*-dihydrodiol intermediates and the derived hydroxyquinolines. 2-Chloroquinoline *cis*-dihydrodiol metabolites were used as precursors in the chemoenzymatic synthesis of enantiopure arene oxide and arene dioxide derivatives of quinoline, in the context of its possible mammalian metabolism and carcinogenicity.

## Introduction

Quinoline and substituted quinolines are widely distributed in the environment as urban particulates, resulting from partial combustion of fossil fuels and tobacco. Quinoline **1** is a mammalian hepatocarcinogen and a bacterial mutagen, with its metabolites binding covalently to DNA. Possible pathways responsible for these biological effects continue to be of interest (Hollstein et al., 1978; Tada et al., 1980, 1982; LaVoie et al., 1983; Agarwal et al., 1986, 1990; Willems et al., 1992; Saeki et al., 1993; Reigh et al., 1996; Suzuki et al., 2000; Dowers et al., 2004; Hakura et al., 2007; Diaz Duran et al., 2015; Matsumoto et al., 2018). Cytochrome P-450 (CYP-450) monooxygenases have been identified as responsible for catalyzing epoxidation and dearomatization of quinolines, during mammalian liver metabolism.

Bacterial cell metabolism can also involve an initial dearomatization step of quinolines, *via* arene dioxygenase-catalyzed *cis*-dihydroxylation, resulting in the isolation of stable, and postulation of transient, azaarene metabolites. This is exemplified by the metabolism of quinoline **1** (X = H), and substituted quinolines, which were studied earlier using different bacterial strains and arene dioxygenase enzyme types (Boyd et al., 1987, 1993, 1998, 2002; Bott et al., 1990; Fetzner et al., 1993; Kaiser et al., 1996; Zia et al., 2016).

**Figure 1:**
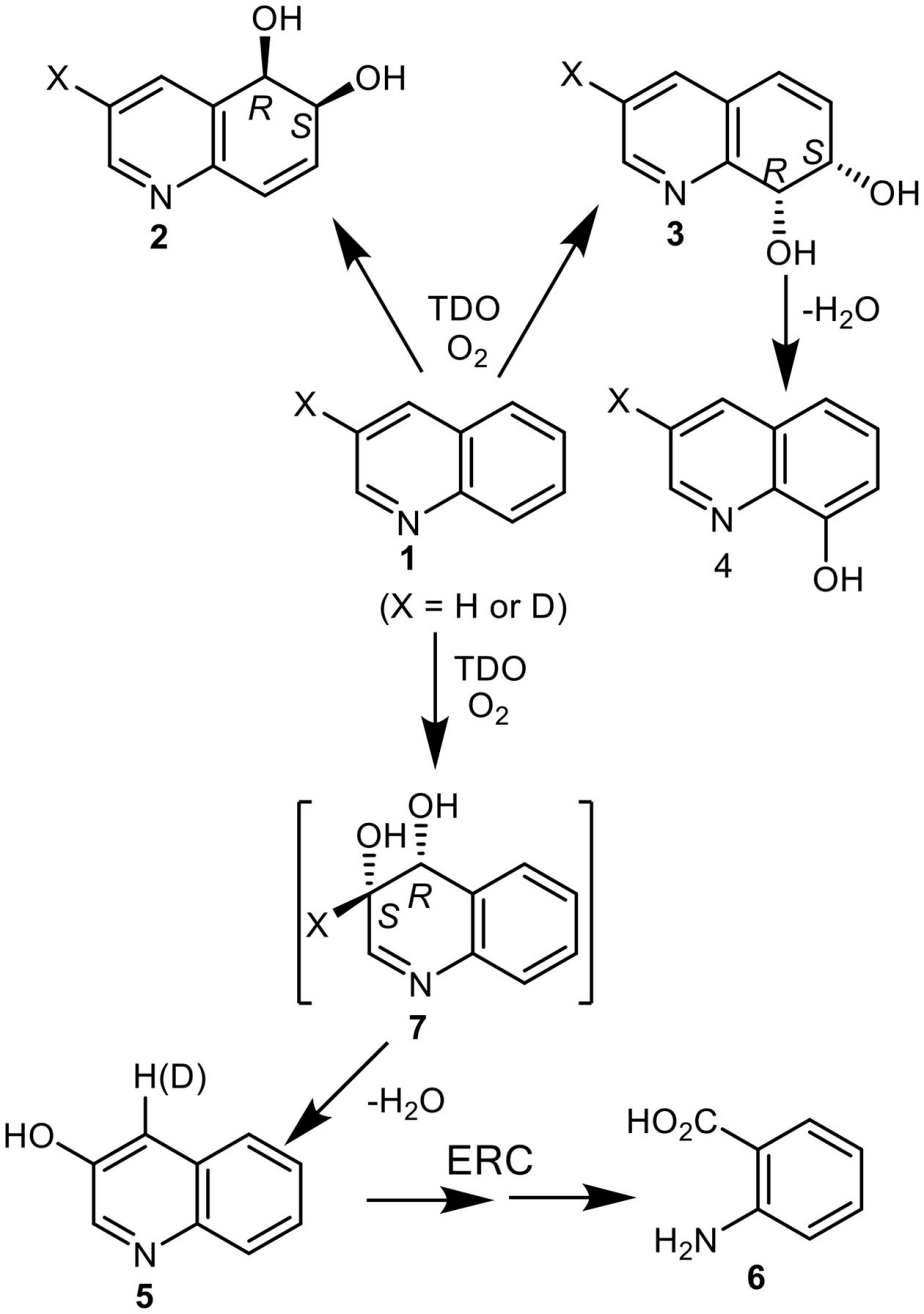
Isolated metabolites, **2–6**, and the postulated transient *cis*-dihydrodiol intermediate **7**, resulting from TDO-catalyzed oxidation of quinoline **1** (X = H or D).

Toluene dioxygenase (TDO), naphthalene dioxygenase (NDO), and biphenyl dioxygenase (BPDO) were among those bacterial enzymes found to catalyze the formation of *cis*-dihydrodiol metabolites and monohydroxylated derivatives of quinoline substrates (Boyd et al., 2002). The UV4 mutant strain of *Pseudomonas putida*, expressing TDO, was used in the biotransformation of quinoline **1** (X = H) to give *cis*-dihydrodiols **2** (33% relative yield) and the less stable *cis*-dihydrodiol **3** (1% relative yield, Figure 1) (Boyd et al., 1987, 1993). As the latter metabolite readily decomposed to 8-hydroxyquinoline **4** (27% relative yield), similar proportions of the *cis*-diols **2** and **3** were assumed to be initially formed from dihydroxylation of the carbocyclic ring of quinoline **1**.

No direct evidence for *cis*-dihydroxylation of the electron-poor pyridyl ring was found during analysis of the biotransformation products (Boyd et al., 1987, 1993). The combined relative yields of the isolated achiral metabolites 3-hydroxyquinoline **5** (13%) and anthranilic acid **6** (27%), were however consistent with the initial formation of the transient *cis*-dihydrodiol **7** as a further major initial metabolite (Figure 1). Formation of 3-hydroxyquinoline **5** could result from spontaneous dehydration of intermediate **7** and of anthranilic acid **6** from enzymatic ring-cleavage (ERC) of 3-hydroxyquinoline **5**. If anthranilic acid **6** and phenol **5** were derived from intermediate *cis*-dihydrodiol **7**, the relative ratio of initially formed *cis*-dihydrodiols diols **2**, **3**, and **7** would be estimated as *ca*. 33:27:40. Further *in silico* support for the formation of transient *cis*-dihydrodiol **7** - the major metabolite of quinoline **1** (TDO as biocatalyst), was obtained from a joint study with a collaborating laboratory using the GOLD molecular docking program (unpublished data). This prompted our interest in employing the Autodock Vina program for the current study.

Indirect evidence for the undetected heterocyclic *cis*-dihydrodiol **7** was acquired from the results of an earlier biotransformation (*P. putida* UV4) of 3-deuterioquinoline **1** (X = 87% D); it yielded 4-deuterioquinolin-3-ol **5** (23% D) along with other metabolites (Figure 1) (Barr et al., 1998). This could be accounted for by the migration and partial retention of deuterium, from the aromatization of the intermediate *cis*-dihydrodiol **7** (X = D, Figure 1) *via* an NIH shift mechanism, as observed during aromatization of the isolated carbocyclic *cis*-dihydrodiols of naphthalene and quinoline **2** and **3**.

*Pseudomonas* strains are among the most common quinoline-degrading bacteria. Monohydroxylation at the C-2 position of quinoline often occurs during *Pseudomonad* biotransformations, to yield 2-hydroxyquinoline, which prefers to exist as the 2-quinolone tautomer (Kaiser et al., 1996). Substitution at the C-2 position could thus, in principle, improve the isolated yields of *cis*-dihydrodiol metabolites resulting from dioxygenase-catalyzed biotransformations of quinoline substrates. It may be a factor in: (i) the excellent isolated yield (80%) of the corresponding 7,8-*cis*-dihydrodiol obtained from NDO-catalyzed biotransformation of 2-cyanoquinoline (Zia et al., 2016) and (ii) the improved isolated yields (35–45%) of *cis*-dihydrodiol metabolites of 2-chloroquinoline (with TDO) and 2-methoxyquinoline (with BPDO) (Boyd et al., 2002), relative to the very low isolated yields of *cis*-dihydrodiols **2** and **3** obtained from quinoline **1** (X = H, <5% using TDO, Figure 1) (Boyd et al., 1993).

**Figure 2:**
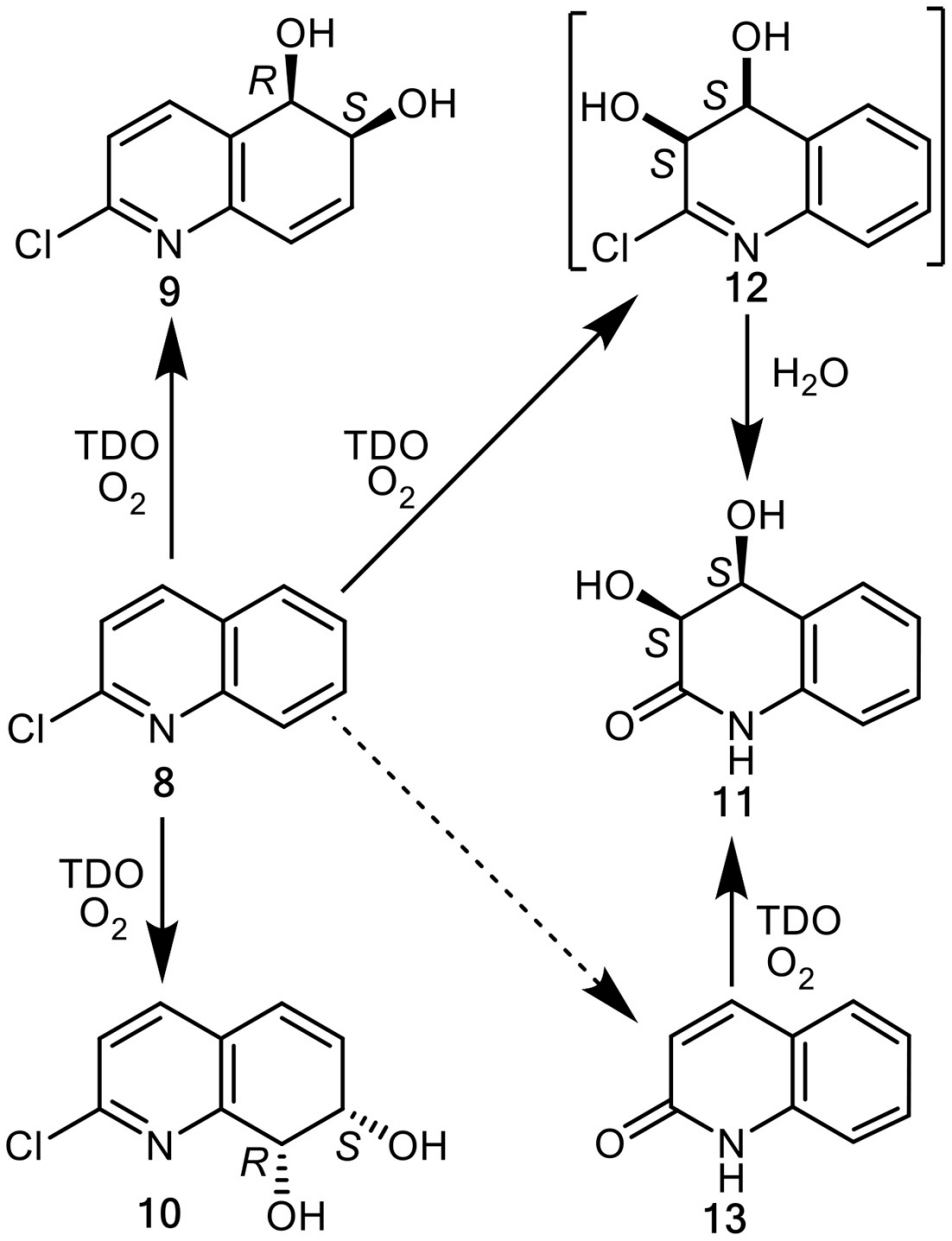
TDO-catalyzed *cis-*dihydroxylation of 2-chloroquinoline **8** and 2-quinolone **13** to yield isolated metabolites **9–11** and postulated transient intermediate **12**.

Biotransformations of 2-chloroquinoline **8**, conducted using *P. putida* UV4 whole cells resulted in the isolation of multigram quantities of *cis*-diol metabolites **9**, **10**, and **11**, possibly *via* intermediate **12**, and traces of 2-quinolone **13** (Figure 2) (Boyd et al., 1998, 2002). Since the enantiopure *cis*-dihydrodiols **9**, **10**, and **11** are more stable, and are available in much higher yields compared with quinoline *cis*-dihydrodiols **2** and **3**, they are herein used in the chemoenzymatic synthesis of enantiopure mammalian metabolites of quinoline **1**.

**Figure 3:**
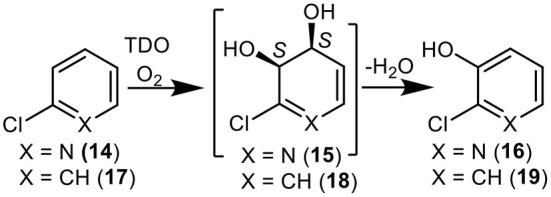
TDO-catalyzed oxidation of 2-chloropyridine **14** and chlorobenzene **17** to yield isolated metabolites **16**, **18**, and **19** and undetected *cis*-dihydrodiol intermediate **15**.

While the dioxygenase-catalyzed *cis*-dihydroxylation of electron-rich furan and thiophene rings has been reported, to give heterocyclic dihydrodiol metabolites (Boyd et al., 2012; Lewis, 2016), little evidence is available for the dearomatization of electron-poor pyridine rings by a similar mechanism. The possibility that TDO-catalyzed *cis*-dihydroxylation of a pyridyl ring was proposed as a result of *P. putida* UV4 biotransformations of: (i) quinoline **1**, to yield 3-hydroxyquinoline **5** *via* unstable intermediate **7** (Figure 1) (Boyd et al., 1987, 1993), (ii) 2-chloroquinoline **8**, to yield *cis*-diol **11** *via* transient intermediate **12** (Figure 2) (Boyd et al., 1998, 2002), and (iii) 2-chloropyridine **14**, to yield 2-chloro-3-hydroxypyridine **16** *via* undetected intermediate **15** (Figure 3) (Garrett et al., 2006). Since none of the *cis*-dihydrodiols **7**, **12**, and **15** could be detected directly, evidence for their intermediacy is sought through the current *in silico* molecular docking studies of TDO with the corresponding substrates, quinoline **1**, 2-chloroquinoline **8** and 2-chloropyridine **14** (Results section).

Major objectives of this study were to: (i) review proposed metabolic pathways of quinoline **1** and 2-chloroquinoline **8** by *P. putida* UV 4 cells, and to compare with TDO docking results (ii) use the isolated *cis*-dihydrodiol metabolites **9** and **10** and their derivatives, in the quest for improved chemoenzymatic synthetic routes to enantiopure arene oxide and dioxide derivatives of quinoline **1**.

## Methods

### Laboratory Studies

^1^H and ^13^C NMR spectra were recorded on Bruker Avance DPX-300 and DPX-500 instruments. Chemical shifts (δ) are reported in ppm relative to SiMe_4_ and coupling constants (*J*) are given in Hertz (Hz). Mass spectra were run at 70 eV, on an AE1-MS90 mass spectrometer updated by VG Autospec, using a heated inlet system. Accurate molecular weights were determined by the peak matching method, with perfluorokerosene as the standard. Elemental microanalyses were carried out on a Perkin-Elmer 2400 CHN microanalyser and IR spectra were recorded in KBr disc or in thin film, using a Perkin-Elmer Spectrum RX1 FT-IR spectrometer. ECD spectra were obtained using a Jasco J-720 instrument and MeCN as solvent. Optical rotations ([α]_D_) measurements (10^−1^ deg cm^2^ g^−1^) were carried out at ambient temperature on a Perkin-Elmer 214 polarimeter and specified solvent concentration (g/100 ml) at sodium D-line (589 nm). Melting points were recorded in degrees Celsius using a Stuart SMP10 melting point apparatus. Column chromatography and preparative layer chromatography (PLC) were performed on Merck Kieselgel type 60 (250–400 mesh) and PF_254/366_, respectively. Merck Kieselgel type 60F_254_ analytical plates were used for TLC.

The structures discussed in this section are found in **Figures 8**, **9**, **11**. Compounds **1**, **4**, **5**, **6**, **8**, **13**, **14**, **16**, **17**, **19**, **22**, **23**, **24** were purchased commercially and *cis*-dihydrodiols (+)-**9** and (+)-**10**, arene oxides (–)-**20** and (+)-**25**, were available from earlier studies (Agarwal et al., 1990; Boyd et al., 1991a, 1994, 2002).

#### (+)-(5*R*,6*S*)-2-Chloro-5,6-dihydroquinoline-5,6-diol 9 (Boyd et al., 1998, 2002)

mp 120–122°C (EtOAc/hexane); [α]_D_ +140 (*c* 0.40, MeOH); ^1^H NMR δ_H_ (500 MHz, CDCl_3_) 2.05 (1H, br s, OH), 2.71 (1H, br s, OH), 4.38 (1H, dd, *J* 5.0, *J* 5.0, H-6), 4.77 (1H, d, *J* 5.0, H-5), 6.45 (1H, dd, *J* 5.0, *J* 9.9, H-7), 6.68 (1H, d, *J* 9.9, H-8), 7.21 (1H, d, *J* 8.0, H-3), 7.85 (1H, d, *J* 8.0, H-4).

#### (+)-(7*S*,8*R*)-2-Chloro-7,8-dihydroquinoline-7,8-diol 10 (Boyd et al., 1998, 2002)

Colorless crystalline solid, mp 112°C (from EtOAc/hexane); [α]_D_ +148 (*c* 0.5, MeOH); ^1^H NMR δ_H_ (500 MHz, CDCl_3_) 2.61 (1H, br s, OH), 4.32 (1H, br s, OH), 4.46 (1H, dd, *J* 5.0, 5.0, H-7), 4.75 (1H, d, *J* 5.0, H-8), 6.30 (1H, dd, *J* 9.6, 5.0, H-6), 6.63 (1H, d, *J* 9.6, H-5), 7.25 (1H, d, *J* 8.0, H-3), 7.42 (1H, d, *J* 8.0, H-4).

#### (–)-(5*R*,6*S*)-5,6-Epoxy-5,6-dihydroquinoline 20 (Agarwal et al., 1990; Boyd et al., 1991a, 1994)

mp 68–71°C (pentane) [lit.rac. (Agarwal et al., 1990) 33–36°C]; [α]_D_ −23 (*c* 0.9, CHCl_3_); ^1^H NMR δ_H_ (300 MHz, CDCl_3_) 4.16 (1H, m, H-6), 4.49 (1H, d, *J* 3.7, H-5), 6.73 (1H, dd, *J* 9.9, 3.8, H-7), 6.98 (1H, d, *J* 9.9, H-8), 7.24 (1H, m, H-3), 7.90 (1H, dd, *J* 7.6.0, 1.4 H-4), 8.62 1H, dd, *J* 4.9.6.0, 1.4 H-2).

#### (+)-(7*S*,8*R*)-7,8-Epoxy-7,8-dihydroquinoline 25 (Agarwal et al., 1990; Boyd et al., 1994)

mp 54–56°C (pentane) (lit.rac (Agarwal et al., 1990) 44–46°C); [α]_D_ +55 (*c* 1.1, CHCl_3_); ^1^H NMR δ_H_ (300 MHz, CDCl_3_) 4.19 (1H, m, H-7), 4.66 (1H, d, *J* 3.7, H-8), 6.51 (1H, dd, *J* 9.6, 3.7, H-6), 6.72 (1H, dd, *J* 9.6,1.7 H-5), 7.31 (1H, dd, *J* 7.7, 4.8, H-3), 7.58 (1H, dd, *J* 7.7.0, 1.3, H-4), 8.53 1H, dd, *J* 4.8, 1.4, H-2).

### Hydrogenation/Hydrogenolysis of *cis*-dihydrodiols (+)-9 and (+)-10 to Yield Tetrahydrodiols (–)-39 and (–)-40

#### (–)-(5*R*,6*S*)-5,6,7,8-Tetrahydroquinoline-5,6-diol 39 (Boyd et al., 1993)

*cis*-Dihydrodiol metabolite **9** (30 mg, 0.15 mmol) was dissolved in MeOH (8 cm^3^) and 10% Pd/C (0.5 mg) was added. The mixture was stirred under a H_2_ atmosphere at atmospheric pressure for 2 h, filtered and concentrated under reduced pressure. Aqueous Na_2_CO_3_ solution (5%) was added to the residue, the mixture saturated with NaCl and then extracted into EtOAc (3 × 10 cm^3^). The combined organic layers were dried (Na_2_SO_4_) and the solvent evaporated to furnish (5*R*,6*S*)-diol **39** (19 mg, 81%); mp 139–140°C (EtOAc) [lit. (Boyd et al., 1993) 138–140°C]; [α]_D_ −7.0 (*c* 0.5, MeOH); ^1^H NMR (300 MHz, CDCl_3_) δ_H_ 1.96 (1H, m, H-7), 2.16 (1H, m, H-7′), 2.85 (1H, m, H-8), 3.09 (1H, m, H-8′), 4.1 (1H, m, H-6), 4.35 (2H, bs, 2 × OH) 4.67 (1H, d, *J* 3.4, H-5), 7.13 (1H, dd, *J* 4.8, 7.7, H-3), 7.79 (1H, dd, *J* 1.1, 7.2, H-4), 8.35 (1H, dd, J 1.5, 4.7, H-2).

#### (–)-(7*S*,8*R*)-5,6,7,8-Tetrahydroquinoline-7,8-diol 40 (Boyd et al., 1993)

Similar treatment of *cis*-dihydrodiol **10** (30 mg, 0.15 mmol) yielded **(**7*S*,8*R*)-diol **40** (21 mg, 81%), mp 137–139°C (EtOAc) [lit. (Boyd et al., 1993) 138–140°C]; [α]_D_ −72.0 (*c* 0.47, MeOH); ^1^H NMR (300 MHz, CDCl_3_) δ_H_ 1.95 (1H, m, H-6), 2.22 (1H, m, H-6′), 2.75 (1H, m, H-5), 3.20 (1H, m, H-5′), 4.32 (1H, m, H-7), 4.92 (1H, d, *J* H-8), 7.17 (1H, dd, *J* 4.7*, J* 7.7, H-3), 7.46 (1H, d, *J* 7.7, H-4), 8.40 (1H, *J* 4.7, H-2).

### Synthesis of (–)-(5*R*,6*R*,7*R*,8*R*)-quinoline-5,6:7,8-dioxide 29 (Boyd et al., 1991b)

Treatment of (–)-(5*R*,6*S*)-5,6-epoxy-5,6-dihydroquinoline **20** (120 mg, 0.8 mmol) in CH_2_Cl_2_ with aqueous sodium hypochlorite in the presence of phase transfer reagent (t-Bu_4_NHSO_4_) and workup, under identical conditions used earlier in the synthesis of racemic *anti*-quinoline dioxide **29**, produced (–)-**29** (51mg, 38%). White crystalline solid (ether-hexane); mp 85–86°C [lit.rac. (Boyd et al., 1991b), 44–46°C]; [α]_D_ −100 (*c* 0.11, CHCl_3_); HRMS calcd for C_9_H_7_NO_2_, 161.0477, M^+^; found, 161.0472; ^1^H NMR (300 MHz, CDCl_3_) δ_H_ 3.73 (1 H, d, *J* 3.9, H-6b), 3.91 (1 H, d, *J* 4.0, H-2a), 4.05 (1 H, m, H-1a), 4.08 (1 H, m, H-1b), 7.28 (1 H, dd, *J* 7.7, 4.8, H-5), 7.73 (1 H, dd, *J* 7.5, 1.5, H-6), 8.53 (1 H, dd, *J* 4.8, 1.5, H-4); ^13^C NMR (125 MHz, CDCl_3_) δ_C_ 50.9, 53.2, 54.7, 55.5, 123.9, 127.7, 138.7, 149.8, 152.4; *m/z* 161 (M^+^, 15%), 132 (100). Electronic CD data: 269 nm (Δε −3.299), 226 nm (Δε −14.97), 197 nm (Δε 11.46).

### Synthesis of (+)-(5*S*,6*S*,7*R*,8*R*)-quinoline-5,6:7,8-dioxide 28 (Boyd et al., 1991b)

(+)-(7*S*,8*R*)-7,8-Epoxy-7,8-dihydroquinoline **25** (120 mg, 0.8 mmol), it was reacted with *N*-bromoacetamide (130 mg, 1.0 mmol) in THF: water (2:1), under identical reaction conditions reported for a racemic arene oxide **25** (Vila et al., 2017). After extraction (CH_2_Cl_2_) drying and concentration, the crude product, the correct NMR spectrum for racemic 6-bromo-5-hydroxy-7,8-epoxy-5,6,7,8-tetrahydroquinoline **41**, was then treated directly with sodium methoxide in THF to yield (+)-(5*S*,6*S*,7*R*,8*R*)-quinoline-5,6:7,8-dioxide **28**. White solid mp 153–156°C [lit.rac. (Boyd et al., 1991b) 138–152°C]; [α]_D_ +25 (*c* 0.2, CHCl_3_); HRMS calcd for C_9_H_7_NO_2_, 161.0477, M^+^; found, 161.0472; ^1^H NMR (500 MHz, CDCl_3_) δ_H_ 3.95–3.99 (2 H, m, H-1a and H-1b), 4.05 (1 H, d, *J* 3.5, H-6b), 4.18 (1 H, d, *J* 3.4, H-2a), 7.38 (1 H, m, H-5), 7.95 (1 H, dd, *J* 7.7, 1.6, H-6), 8.65 (1 H, dd, *J* 4.9, 1.6,H-4); *m/z* 161 (M^+^, 15%), 132 (100). Electronic CD data: 274 nm (Δε 15.93), 268 nm (Δε 14.31), 246 nm (Δε −3.425), 192 nm (Δε −16.08).

### *Cis*-dihydroxylation of (+)-*cis*-diol 9 [and 10] and Acetylation to Yield Tetrahydroacetates, (+)-44, (–)-44 [(+)-42 and (+)-45]

To a stirred solution of (+)-*cis*-dihydrodiol **9** (200 mg, 1.02 mmol) and trimethylamine-*N*-oxide dihydrate (150 mg, 1.34 mmol) in CH_2_Cl_2_ (50 ml) was added a catalytic amount of OsO_4_ and the mixture stirred overnight at room temperature. After the addition of a 10% solution of sodium metabisulfite (2 ml), the reaction mixture was allowed to stir for another 0.5 h and then concentrated under reduced pressure to give a crude mixture of inseparable *syn*- and *anti*-tetraols. The mixture was acetylated by heating (75–80°C), overnight, with excess of acetic anhydride (*ca*. 2 ml) in pyridine (2 ml) solution. Concentration of the reaction mixture in *vacuo*, yielded a crude mixture (3:1) of *anti*-**45** and *syn*-**44** tetraacetates. After initial purification of the mixture, using flash chromatography (silica gel, 3% MeOH in CHCl_3_), followed by multiple elution PLC (20% EtOAc in hexane) separation gave major *anti*-tetraacetate (+)-**45** and minor *syn*-tetraacetate (–)-**44**.

#### (+)-(5*R*,6*R*,7*R*,8*R*)-5,6,7,8-Tetraacetoxy-2-chloro-5,6,7,8-tetrahydroquinoline 45

White crystalline solid (121 mg, 30%); (*R*_f_ 0.3, 30% EtOAc in hexane); mp 87–88°C (from CHCl_3_); [α]_D_ +14.6 (*c* 0.83, CHCl_3_); (Found: C, 50.6; H, 4.7; N, 3.3. C_17_H_18_ClNO_8_ requires C, 51.1; H, 4.5; N, 3.5%); ^1^H NMR (500 MHz, CDCl_3_) δ_H_ 2.07 (3H, s, OMe), 2.08 (1H, s, OMe), 2.11 (1H, s, OMe), 2.13 (1H, s, OMe), 5.63 (1 H, dd, *J* 10.1, 3.9, H-6), 5.73 (1 H, dd, *J* 10.1, *J* 4.1, H-7), 6.34 (1 H, d, *J* 3.9, H-5), 6.39 (1 H, d, *J* 4.1, H-8), 7.35 (1 H, d, *J* 8.3, H-3), 7.63 (1 H, d, *J* 8.3, H-4); ^13^C NMR (CDCl_3_, 125 MHz) δ_C_ 20.5, 20.6, 20.7, 20.8, 66.7, 67.1, 67.3, 67.6, 124.9, 127.1, 139.1, 151.5, 152.3, 169.7, 169.8, 169.9, 170.2, *m/z* 339 (M^+^, 15%, ^35^Cl), 43 (100); ν_max_/cm^−1^: 1,741 (C=O).

#### (–)-(5*R*,6*R*,7*S*,8*S*)-5,6,7,8-Tetraacetoxy-2-chloro-5,6,7,8-tetrahydroquinoline 44

White crystalline solid (53 mg, 13%); (*R*_f_ 0.25, 30% EtOAc-hexane); mp 177–178°C (CHCl_3_); [α]_D_ −20 (*c* 0.69, CHCl_3_); (Found: C, 50.8; H, 4.3; N, 3.6. C_17_H_18_ClNO_8_ requires C, 51.1; H, 4.5; N, 3.5%); HRMS calcd for C_17_H_18_ClNO_8_, 399.0721, M^+^; found, 399.0727; ^1^H NMR (500 MHz, CDCl_3_) δ_H_ 2.07–2.17 (12 H, s, 4 × COCH_3_), 5.58 (1 H, dd, *J* 4.5, 2.2, H-7), 5.62 (1 H, dd, *J* 2.2, 4.7, H-5), 6.22 (1 H, d, *J* 4.4, H-8), 7.34 (1 H, d, *J* 8.2, H-3), 7.61 (1 H, d, *J* 8.2, H-4); ^13^C NMR (125 MHz, CDCl_3_) δ_C_ 19.6, 19.7, 19.8, 20.0, 65.6, 66.1, 66.3, 66.8, 124.1, 126.1, 139.3, 150.5, 151.3, 168.7, 168.8, 168.9, 169.1; *m/z* 399 (M^+^, 16%, ^35^Cl), 43 (100); ν_max_/cm^−1^: 1,741 (C=O).

#### (+)-(5*S*,6*S*,7*R*,8*R*)-5,6,7,8-Tetraacetoxy-2-chloro-5,6,7,8-tetrahydroquinoline 44

Employing a similar osmylation/acetylation procedure, used for metabolite (+)-**9**, to *cis*-dihydrodiol (+)-**10** gave a mixture (1:3) of the opposite *syn*-tetraacetate enantiomer, (+)-**44**, [α]_D_ +19.0 (*c* 0.74, CHCl_3_) and the *anti*-tetraacetate enantiomer (+)-**45**.

### Hydrolysis of Chlorotetraacetates (–)-44, (+)-44, and (+)-45 to Yield Chlorotetrols (–)-42, (+)-42, and (+)-43

#### (–)-(5*R*,6*R*,7*S*,8*S*)-2-Chloro-5,6,7,8-tetrahydro-5,6,7,8-quinoline tetraol 42

A solution of tetraacetate (–)-**44** (300 mg, 0.75 mmol) in MeOH (30 ml) was saturated with NH_3_ gas at 0°C and was left overnight in an ice bath. The solvent was evaporated under reduced pressure, and the ammonium acetate side product was removed by sublimation under high vacuum. Crystallization of the residue from a mixture of acetone-MeOH-CHCl_3_ gave pure tetrol (–)-**42** (130 mg, 75%), white needles; mp 153–154°C; [α]_D_ −8 (*c* 0.68, MeOH); (Found: C, 45.7; H, 4.3; N, 5.8. C_9_H_10_NO_4_Cl requires C, 46.7; H, 4.3; N, 6.1%); ^1^H NMR (500 MHz, D_2_O) δ_H_ 4.17 (1 H, m, *J* 2.1, 4.4, H-7), 4.19 (1 H, m, *J* 2.0, 4.13, H-6), 4.68 (1 H, d, *J* 4.4, H-8), 4.77 (1 H, d, *J* 4.13, H-5), 7.45 (1 H, d, *J* 8.3, H-3), 7.93 (1 H, d, *J* 8.3, H-4).

#### (+)-(5*S*,6*S*,7*R*,8*R*)-2-Chloro-5,6,7,8-tetrahydro-5,6,7,8-quinoline tetrol 42

Similar treatment of tetraacetate (+)**-44** gave pure tetrol (+)-**42** [α]_D_ +7 (*c* 0.44, MeOH).

#### (+)-5*R*,6*R*,7*R*,8*R*)-2-Chloro-5,6,7,8-tetrahydro-5,6,7,8-quinoline tetrol 43

Tetraacetate (+)-**45** (1.35 g, 3.41 mmol) was deprotected and crystallized in a similar manner to compound (-)-**44** to yield pure tetraol (+)-**43** (600mg, 76%), white crystals; mp 189-192 °C; [α]_D_ +14.6 (*c* 0.57, MeOH); (Found: C, 46.6; H, 4.1; N, 6.0. C_9_H_10_NO_4_Cl requires C, 46.7; H, 4.3; N, 6.1%); Found: M^+^ 231.03090, C_9_H_10_ClNO_4_ requires 231.02984; ν_max_/cm^−1^: 3391 (OH); ^1^H NMR (500 MHz, D_2_O) δ_H_ 4.25 (1 H, dd, *J* 5.6, *J* 3.9, H-6), 4.30 (1 H, dd, *J* 5.6, *J* 4.0, H-7), 4.91 (1 H, d, *J* 4.0, H-8), 5.00 (1 H, d, *J* 3.9, H-5), 7.57 (1 H, d, *J* 8.3, H-3), 7.97 (1 H, d, *J* 8.3, H-4); ^13^C NMR (125 MHz, D_2_O) δ_C_ 67.9, 69.0, 69.1, 70.3, 125.6, 131.2, 142.5, 151.5, 154.6; *m/z* 231 (M^+^, 5%, ^35^Cl), 213 (34%), 171 (100).

### Hydrogenolysis of Chlorotetrols (–)-42, (+)-42, and (+)-43 to Yield Tetrols (–)-46, (+)-46, and (–)-48

#### (+)-(5*R*,6*R*,7*S*,8*S*)-5,6,7,8-Tetrahydro-5,6,7,8-quinoline tetraol 46

A solution of chlorotetraol (–)**-42** (200 mg, 0.86 mmol) in MeOH (20 ml) containing 10% Pd/C (50 mg) was stirred overnight at room temperature, under H_2_ atmosphere at 1 atm. pressure. The reaction mixture was basified (NH_3_ solution), the catalyst was filtered off and the filtrate concentrated under reduced pressure to yield tetraol (–)-**46** (120 mg, 71%), white solid (MeOH); mp 164–165°C (MeOH); [α]_D_ +25 (*c* 0.5, MeOH); (Found: C, 54.3; H, 5.3; N, 6.6. C_9_H_11_NO_4_ requires C, 54.8; H, 5.6; N, 7.1%); ^1^H NMR (500 MHz, D_2_O) δ_H_ 4.28 (1 H, dd, *J* 4.3, 1.4, H-7), 4.34 (1 H, dd, *J* 4.0, 1.4, H-6), 4.88 (1 H, d, *J* 4.3, H-8), 4.93 (1 H, d, *J* 4.0, H-5), 7.56 (1 H, dd, *J* 7.9, 4.5, H-3), 8.10 (1 H, d, *J* 7.9, H-4), 8.62 (1 H, d, *J* 4.5, H-2).

#### (–)-(5*S*,6*S*,7*R*,8*R*)-5,6,7,8-Tetrahydro-5,6,7,8-quinoline tetraol 46

A similar hydrogenolysis process on compound (+)**-44** yielded tetraol (–)-**45**, [α]_D_ −23 (*c* 0.4, MeOH).

#### (–)-(5*R*,6*R*,7*R*,8*R*)-5,6,7,8-Tetrahydro-5,6,7,8-quinoline tetrol 48

Hydrogenolysis of tetrol **(+)-43** (0.2 g, 0.86 mmol) gave tetraol **(**–**)-48**, (0.12 g, 71%); mp 186-187 °C (MeOH); [α]_D_ −104 (*c* 0.56, pyridine); (Found: C, 53.6; H, 5.8; N, 6.6. C_9_H_11_NO_4_ requires C, 54.8; H, 5.6; N, 7.1%); ^1^H NMR (500 MHz, D_2_O) δ_H_ 4.33 (1 H, dd, *J* 6.7, *J* 6.8, H-7), 4.38 (1 H, dd, *J* 6.8, *J* 6.5, H-6), 5.05 (1 H, d, *J* 6.7, H-8), 5.09 (1 H, d, *J* 6.5, H-5), 7.62 (1 H, dd, *J* 7.8, *J* 4.0, H-3), 8.06 (1 H, d, *J* 4.0, H-2), 8.69 (1 H, d, *J* 7.8, H-4); *m/z* 197 (M^+^, 7.5%), 137 (100).

### Synthesis of Dibromohydroxyacetates (–)-47, (+)-47 and Dibromodiacetate 49 From tetrols (+)-46, (–)-46, and (–)-48

#### (–)-(5*S*,6*S*,7*R*,8*R*)-5,8-Dibromo-6-hydroxy-5,6,7,8-tetrahydro-7-quinolinyl acetate 47

To a stirred solution of tetrol (+)-**46** (100 mg, 0.51 mmol) in dry CH_3_CN (5 ml) maintained at ice temperature, was added 1-bromocarbonyl-1-methylethyl acetate (0.15 ml, 1.04 mmol) and the stirring continued (2.5 h) at room temperature. The reaction mixture was concentrated *in vacuo*, the concentrate taken up into ether (20 ml) and washed repeatedly with 2.5% NaHCO_3_ solution until the aqueous layer was basic. The combined aqueous layer was back extracted with ether and the combined ether extract dried (Na_2_SO_4_) and concentrated under reduced pressure. Purification of the crude product by PLC yielded compound (–)-**47** (80 mg, 65%), pale yellow oil; (*R*_f_ 0.25, 55% EtOAc-hexane); [α]_D_ −31 (*c* 0.54, CHCl_3_); HRMS calcd for C_11_H_10_NO_3_Br, 282.9844, (M – HBr)^+^; found, 282.9839; ^1^H NMR (500 MHz, CDCl_3_) δ_H_ 2.08 (3 H, s, COCH_3_), 4.90 (1 H, dd, *J* 8.3, 2.3, H-6), 5.30 (1 H, d, *J* 4.0, H-8), 5.40 (1 H, d, *J* 8.3, H-5), 5.75 (1 H, dd, *J* 4.1, *J* 2.3, H-7), 7.32 (1 H, dd, *J* 8.9, H-4), 8.00 (1 H, d, *J* 8.9, H-4), 8.59 (1 H, d, *J* 5.6, H-2); ^13^C NMR (125 MHz, CDCl_3_) δ_C_ 20.9, 46.3, 51.5, 70.3, 74.3, 123.9, 130.2, 139.6, 150.3, 151.2, 169.9; *m/z* 285 (M-HBr)^+^, ^79^Br, 15%), 287 (M^+^-HBr, ^81^Br, 15%), 242 (100).

#### (+)-(5*R*,6*R*,7*S*,8*S*)-5,8-Dibromo-6-hydroxy-5,6,7,8-tetrahydro-7-quinolinyl acetate 47

A similar treatment procedure on tetrol (–)**-46**, yielded compound (+)-**47** [α]_D_ +30 (*c* 0.4, CHCl_3_).

#### (5*S*,6*S*,7*S*,8*S*)-6,7-(Diacetoxy)-5,6-dibromo-5,6,7,8-tetrahydroquinoline 49

Similar treatment of (–)-tetraol **48** (0.15 g, 0.78 mmol) with 1-bromocarbonyl-1-methylethyl acetate gave dibromodiacetate **49** (0.21 g, 65%), pale yellow syrup; (*R*_f_ 0.7, 55% EtOAc-hexane); (Found: M^+^-HBr, 324.996063. C_13_H_12_NO_4_Br requires M^+^, 324.994969); ^1^H NMR (500 MHz, CDCl_3_) δ_H_ 2.12 (3 H, s, COCH_3_), 2.15 (3 H, s, COCH_3_), 5.36 (1 H, d, *J* 6.1, H-8), 5.40 (1 H, d, *J* 7.5, H-5), 5.52 (1 H, m, H-6), 5.65 (1 H, m, H-7), 7.35 (1 H, dd, *J* 8.0, *J* 4.7, H-3), 7.99 (1 H, d, *J* 8.0, H-4), 8.65 (1 H, d, *J* 4.3, H-2); ^13^C NMR (125 MHz, CDCl_3_) δ_C_ 20.7, 23.9, 34.2, 46.4, 47.4, 58.8, 73.8, 124.2, 128.9, 134.1, 150.4, 150.6, 169.2, 169.3; *m/z* 325 (M^+^-HBr, 50%), 79 (100).

### Synthesis of Quinoline-5,6: 7,8-dioxide (–)-28 and (+)-28 From 5,8-dibromo-6-hydroxy-5,6,7,8-tetrahydro-7-quinolinyl acetate (–)-47 and (+)-47

#### (–)-(5*R*,6*R*,7*S*,8*S*)-Quinoline-5,6:7,8-dioxide 28

A solution of bromohydrin (–)-**47** (80 mg, 0.22 mmol) in THF (10 ml) was stirred at 0°C with an excess of anhydrous sodium methoxide (250 mg) for 1 h and at room temperature for another 1 h. After removal of the THF under reduced pressure ice-cold water (10 ml) was added to the residue and the aqueous mixture extracted with ether. The extract was dried (Na_2_SO_4_) concentrated and the crude product purified by PLC (*R*_f_ 0.2, 60% EtOAc in hexane) to give *syn*-quinoline dioxide (–)-**28** (25 mg, 70%), white solid (CH_2_Cl_2_-ether-hexane); mp 153–156°C (lit (Boyd et al., 1991b) rac. mp 138–152°C); [α]_D_ −26 (*c* 0.2, CHCl_3_); HRMS calcd for C_9_H_7_NO_2_, 161.0477, M^+^; found, 161.0472; ^1^H NMR (500 MHz, CDCl_3_) δ_H_ 3.95–3.99 (2 H, m, H-1a, and H-1b), 4.05 (1 H, d, *J* 3.5, H-6b), 4.18 (1 H, d, *J* 3.4, H-2a), 7.38 (1 H, m, H-5), 7.95 (1 H, dd, *J* 7.7, 1.6, H-6), 8.65 (1 H, dd, *J* 4.9, 1.6,H-4); *m/z* 161 (M^+^, 15%), 132 (100). Electronic CD data: 274 nm (Δε 15.93), 268 nm (Δε 14.31), 246 nm (Δε −3.425), 192 nm (Δε −16.08).

#### (+)-(5*S*,6*S*,7*R*,8*R*)-Quinoline-5,6:7,8-dioxide 28

Dibromoacetate (–)**-46** yielded quinoline dioxide **28** [α]_D_ +25 (*c* 0.2, CHCl_3_).

### Synthesis of (5*R*,6*R*,7*R*,8*R*)-Quinoline-5,6:7,8-dioxide 29 and (5*R*,6*S)-*8-bromoquinoline-5,6-oxide 50 From diacetoxy-5,6-dibromo-5,6,7,8-tetrahydroquinoline 49

Similar treatment of dibromodiacetate **49** (200 mg, 0.48 mmol) with sodium methoxide gave a mixture of two products. PLC separation (50% EtOAc-hexane) yielded quinoline dioxide (–)-**29** (46 mg, 60%) and bromoquinoline oxide (–)-**50** (16 mg, 15%).

#### (–)-(5*R*,6*R*,7*R*,8*R*)-Quinoline-5,6:7,8-dioxide 29

*R*_f_ 0.4 (EtOAc- hexane); white solid; mp 85–86°C (ether-hexane) [lit. Boyd et al., 1991b rac.m.p. 44–46°C]; [α]_D_ −100 (*c* 0.11, CHCl_3_); (Found: M^+^ 161.047158. C_9_H_7_NO_2_ requires 161.047679); ^1^H NMR (300 MHz, CDCl_3_) δ_H_ 3.73 (1 H, d, *J* 3.9, H-6b), 3.91 (1 H, d, *J* 4.0, H-2a), 4.05 (1 H, m, H-1a), 4.08 (1 H, m, H-1b), 7.28 (1 H, dd, *J* 7.7, *J* 4.8, H-5), 7.73 (1 H, dd, *J* 7.5, *J* 1.5, H-6), 8.53 (1 H, dd, *J* 4.8, *J* 1.5, H-4); ^13^C NMR (125 MHz, CDCl_3_) δ_C_ 50.9, 53.2, 54.7, 55.5, 123.9, 127.7, 138.7, 149.8, 152.4; *m/z* 161 (M^+^, 15%), 132 (100). Electronic CD data: 269 nm (Δε −3.30), 226 nm (Δε −14.97), 197 nm (Δε 11.46).

#### (–)-(5*R*,6*S*)-8-Bromo quinoline-5,6-oxide 50

*R*_f_ 0.6 (50% EtOAc-hexane); white solid; mp 107–108°C (ether- pet. ether), [α]_D_ −104 (*c* 0.5, CHCl_3_), (Found: C, 48.9; H, 2.2; N, 6.0. C_9_H_6_NOBr requires C, 48.3; H, 2.7; N, 6.2); ^1^H NMR (500 MHz, CDCl_3_) δ_H_ 4.16 (1 H, dd, *J* 4.3, *J* 3.9, H-1a), 4.53 (1 H, d, *J* 3.8, H-7b), 7.25 (1 H, d, *J* 4.3, H-2), 7.35 (1 H, dd, *J* 7.7, *J* 4.9, H-6), 7.95 (1 H, d, *J* 7.6, H-7), 8.78 (1 H, d, *J* 4.9, H-5); ^13^C NMR (125 MHz, CDCl_3_) δ_C_ 53.7, 56.6, 123.3, 128.8, 129.1, 132.1, 137.8, 149.9, 150.0; *m/z* 223 (M^+^, ^79^Br, 60%), 225 (M^+^, ^81^Br, 60%), 89 (100); Electronic CD data: 302 nm (Δε ^_^1.92), 276 nm (Δε 2.48), 239 nm (Δε ^_^8.93), 233 nm (Δε −8.60), 225 nm (Δε −9.38).

### Docking Process

All small molecule structures were created in.pdb format using UCSF Chimera (University of California). The crystal structures of TDO (PDB ID: 3en1) and NDO (1o7M) were obtained from the RCSB Protein Data Bank. The toluene contained in the TDO crystal structure was removed using UCSF Chimera. The dioxygen molecule was added to the iron prosthetic group of TDO (Fe, His222, His228, Asp376) from the iron prosthetic group (Fe, dioxygen, His-208, His-213, and Asp-362) of the NDO crystal structure using the “super” function of PyMol 2.4.0 to overlay the two partial structures at the position of 3en1 and copy the dioxygen atoms to the 3en1 model. All structures in.pdb format were then stripped of water molecules and converted to .pdbqt format using AutoDock Tools 1.5.6 (Scripps Research Institute).

The docking was performed using AutoDock Vina 1.1.2 (Scripps Research Institute) with the following configuration:

$$\begin{array}{l} \text{ center\_x}=46.46\\ \text{ center\_y}=120.49\\ \text{ center\_z}=201.877\\ \text{ size\_x}=16\\ \text{ size\_y}=16\\ \text{ size\_z}=16\\ \text{exhaustiveness}=100 \end{array}$$

This configuration includes the amino acids within 6 Å of the toluene present in the 3en1 crystal structure: Gln215, Phe216, Met220, His222, Ala223, His228, Leu 272, Ile276, Ile232, Val309, His311, Leu321, Ile324, Phe366, Phe372, and Asp376. Only Met220 is not shown in the docking structures as it blocked the view on the docking orientation and was considered irrelevant for catalytic purposes.

Based on earlier publications and current understanding of the binding site and catalytic mechanism, His 222 is involved in edge-to-face binding of the substrate to direct a planar orientation, presenting a face to the active site. The probability for the methyl group of the toluene substrate to be oriented toward Phe216 is reduced by this residue, thus resulting in the observed enantiomeric excess. His311 and Asn215 are considered to be essential for the catalytic mechanism, as His311 is a H-acceptor and Asn215 is considered to be part of a water channel.

The docking model was assessed by docking toluene and comparing obtained orientations to the toluene position in the crystal structure. A total of nine orientations were obtained ranging from −5.3 to −4.2 kcal/mol^−1^. Three orientations emerged that present the 2,3-double bond to the dioxygen for dihydroxylation (see Supplementary Figures S-13A–C). These orientations showed minor differences in position compared to the orientation observed in the crystal structure (1–1.2 Å) and compared to each other. The difference in position between the docked and crystal structure was probably affected by the docking with dioxygen, as the crystal structure does not contain dioxygen, allowing the toluene to be closer to the iron prosthetic group. Two identical orientations emerged (see Supplementary Figure S-13D) which would result in the opposite enantiomer being formed. Given the enantiomeric excess of >98% of the dihydroxylated product of toluene, it appears unlikely that this orientation is readily dihydroxylated.

## Results

### Molecular Docking of TDO With Quinoline 1, 2-Chloroquinoline 8, 2-Chloropyridine 14 and Chlorobenzene 17

Molecular docking studies of TDO-catalyzed *cis*-dihydroxylations of mono- and di-substituted benzene substrates were conducted in other laboratories, using the GOLD program (Vila et al., 2016a,b, 2017). AutoDock and AutoDock Vina programs have similarly been used in our laboratories, to study TDO-catalyzed *cis*-dihydroxylation of other types of monocyclic arenes, e.g., substituted phenols and anilines and tricyclic heteroarenes, e.g., dibenzofuran and dibenzothiophene (Hoering et al., 2016; Boyd et al., 2017, 2019). Relative free binding energies (kcal/mol^−1^) and relative proximity of arene bonds to dioxygen, within the TDO active site (Å), were determined by applying the AutoDock Vina program to monocyclic arenes (toluene, chlorobenzene) and mono- and bicyclic- azarenes in the current study.

**Figure 4:**
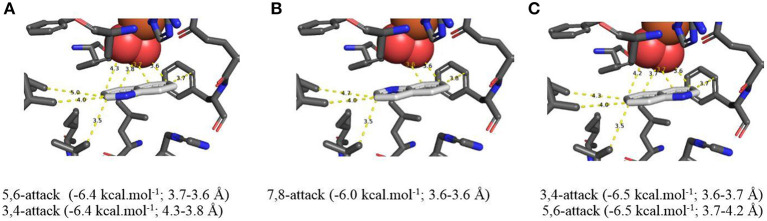
**(A–C)** TDO docking orientations of quinoline **1** (X = H) consistent with preferential *cis*-dihydroxylation at 5,6- (−6.4 kcal·mol^−1^, **A**), 7,8- (−6.0 kcal·mol^−1^, **B**), and 3,4-bonds (−6.5 kcal·mol^−1^, **C**). Hydrophobic substrate coordinating amino acids in the active site shown include Phe 326 and Val 309, The iron atom shown is coordinated by His 222, 228, and Asp 376. The positioning of the docked substrate relative to the positioned bound oxygen is consistent with experimentally observed product regio specificity.

Using the AutoDock Vina docking model, to indicate binding energy (kcal/mol^−1^) and bond proximity (Å) values of TDO with quinoline substrate **1** (X = H), *cis*-dihydroxylation of the carbocyclic ring was predicted to occur preferentially at the 5,6- (Figure 4A, −6.4 kcal/mol^−1^, 3.6–3.7 Å) and 7,8- bonds (Figure 4B, −6.0 kcal/mol^−1^, 3.6 Å). This would be consistent with formation of similar quantities of the 5*R*,6*S* and 7*S*,8*R cis*-dihydrodiols **2** and **3**, respectively. When the isolated yields of *cis*-dihydrodiol **3** and the derived 8-hydroxyquinoline **4** are combined, the relative proportions of originally formed *cis*-dihydrodiol regioisomers **2**:**3** were similar and 5*R*,6*S* and 7*S*,8*R* configurations were firmly established (Boyd et al., 1987, 1993).

Alternative *in silico* orientations of quinoline **1** within the TDO active site (Figure 4C, −6.5 kcal/mol^−1^, 3.7–4.2 Å), and (Supplementary Figure 4D, −5.8 kcal/mol^−1^, 3.8–4.2 Å), would lead to the incorrect 5*S*,6*R* and 7*R*,8*S* absolute configurations for isolated metabolites **2** and **3**, respectively. Although these orientations had similar binding energies, compared with Figures 4A,B it was presumed that the larger distances between the relevant bonds and proximate dioxygen atom were the determining factors.

Support for the intermediacy of the undetected heterocyclic ring *cis*-dihydrodiol **7** was provided by the AutoDock Vina-derived prediction that TDO-catalyzed *cis*-dihydroxylation could occur at the 3,4-bond of the pyridine ring to yield transient intermediate (3*S*,4*R*)-**7** (Figure 4C, −6.5 kcal/mol^−1^, 3.6–3.7 Å). The opposite (3*R*,4*S*) enantiomer **7** might also be expected from the orientation of quinoline **1** in Figure 4A. While a similar binding energy (−6.4 kcal/mol^−1^) is shown in Figure 4A, this orientation may be less favored, due to a larger distance from the nearest dioxygen atom (3.8–4.3 Å).

**Figure 5:**
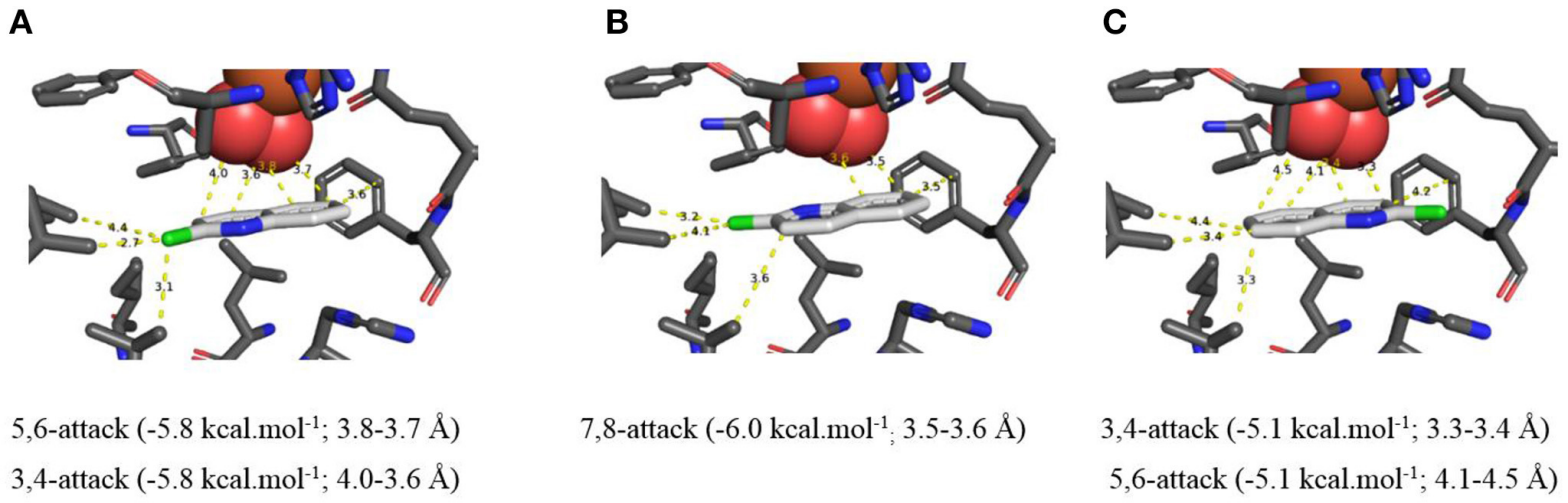
**(A–C)** TDO docking of 2-chloroquinoline **8** with *cis*-dihydroxylation at a 5,6- (**A**, −5.8 kcal·mol^−1^), 7,8- (**B**, −6.0 kcal·mol^−1^), and 3,4-bond (**C**, −5.1 kcal·mol^−1^). Hydrophobic substrate coordinating amino acids in the active site shown include Phe 326 and Val 309. The iron atom shown is coordinated by His 222, 228, and Asp 376. The positioning of the docked substrate relative to the positioned bound oxygen is consistent with experimentally observed product regio specificity.

*In silico* data collected using the Autodock Vina TDO-docking program with 2-chloroquinoline **8** as substrate, similarly led to the prediction that *cis*-dihydroxylation should occur at the 5,6-position, to give (5*R*,6*S*)-dihydrodiol **9** (Figure 5A, −5.8 kcal/mol^−1^, 3.7–3.8 Å) and at the 7,8-bond to form (7*S*,8*R*)-dihydrodiol **10** (Figure 5B, −6.0 kcal/mol^−1^, 3.5–3.6 Å). Alternative docking orientations could also result in *cis*-dihydroxylation at the 5,6 bond (Figure 5C, −5.1 kcal/mol^−1^, 4.1–4.5 Å) and the 7,8 bond (Supplementary Figure 5D, −6.2 kcal/mol^−1^, 4.1–4.5 Å), but the resulting *cis*-diol metabolites **9** and **10** would have the opposite absolute configurations to the isolated metabolites (5*R*,6*S* and 7*S*,8*R*, respectively). The orientations in Figure 5C and Supplementary Figure 5D were again assumed to be less favored, due to the larger distances between substrate and nearby dioxygen atoms. Thus, the absolute configurations of the quinoline *cis*-dihydrodiols (**2**, **3**, **9**, **10**) predicted by *in silico* results were identical to those found earlier for metabolites isolated from *P. putida* UV4 biotransformations (Boyd et al., 1987, 1993, 1998, 2002).

**Figure 6:**
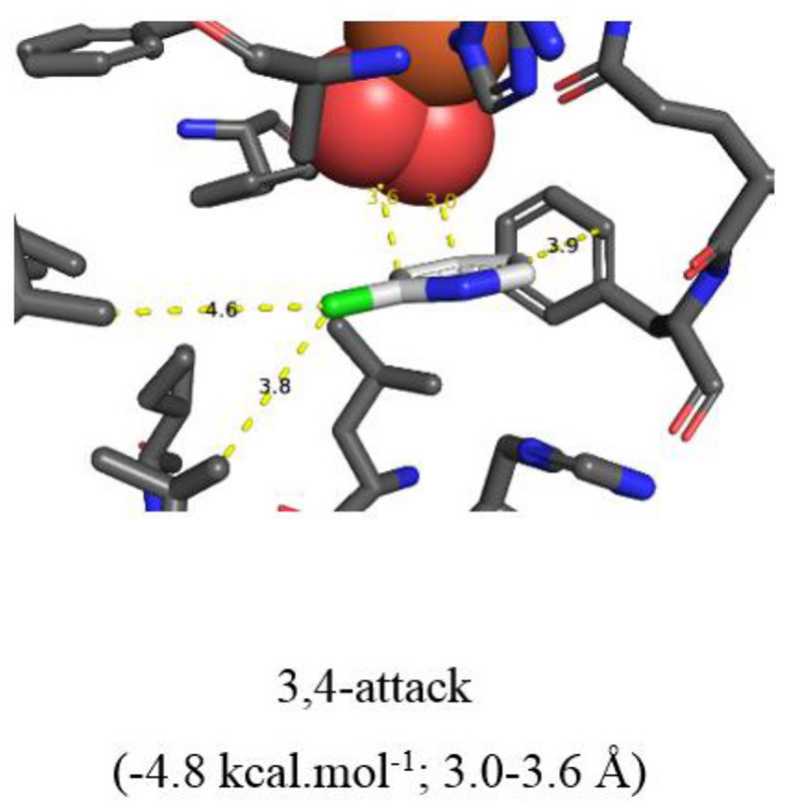
TDO docking of 2-chloropyridine **14** with *cis*-dihydroxylation at a 3,4-bond (−4.8 kcal·mol^−1^, this figure) and of chlorobenzene **17** at a 2,3 bond (−4.9 kcal·mol^−1^, **Figure 7**). Hydrophobic substrate coordinating amino acids in the active site shown include Phe 326 and Val 309. The iron atom shown is coordinated by His 222, 228, and Asp 376. The positioning of the docked substrate relative to the positioned bound oxygen is consistent with experimentally observed product regio specificity.

**Figure 7:**
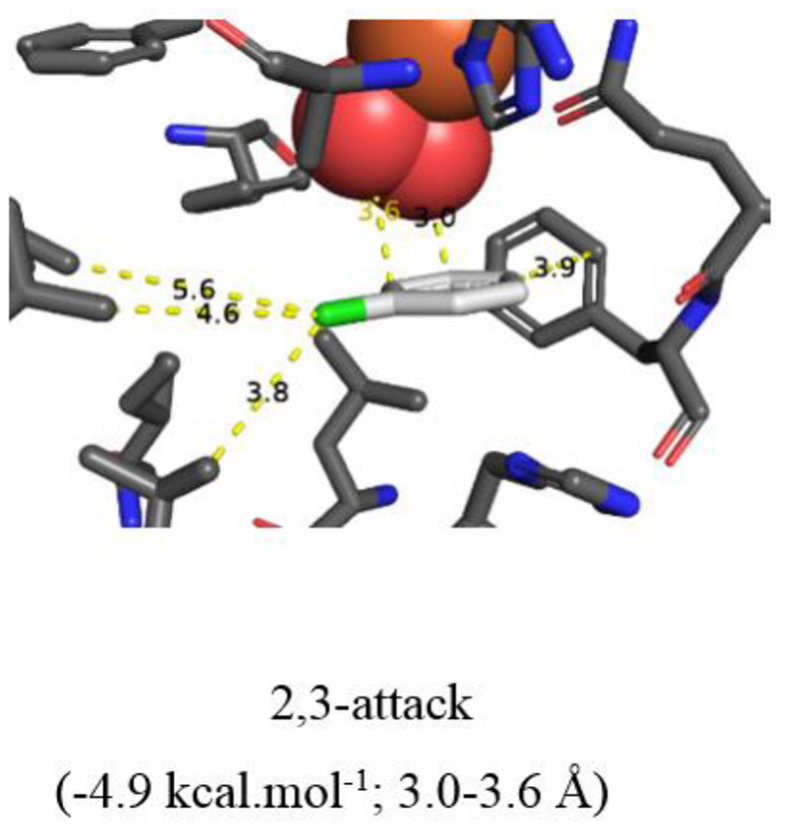
Same as Figure 6.

More evidence for the TDO-catalyzed *cis*-dihydroxylation of a pyridyl ring was sought by docking studies of 2-chloropyridine **14** and comparison with chlorobenzene **17**. Substrates **14** (Figure 6, −4.8 kcal/mol^−1^, 3.0–3.6 Å) and **17** (Figure 7, −4.9 kcal/mol^−1^, 3.0–3.6 Å) were found to adopt similar orientations, within the TDO active site, leading to the prediction that the derived (*S, S*) enantiomers of *cis*-dihydrodiols **15** and **18**, would be formed preferentially. The (2*S*,3*S*) configuration for isolated metabolite **18**, and its dehydration to yield phenol **16**, was already established (Ziffer et al., 1977). The undetected intermediate **15**, derived from 2-chloropyridine **14**, was assumed to dehydrate more readily to yield the isolated hydroxypyridine **16** (Garrett et al., 2006). These docking results thus support the mechanism shown in Figure 3 (**14**→**15**→**16**).

Based on the AutoDock Vina results, the orientations within the TDO active site, favored by heterocyclic arene substrates, e.g., **1** (X = H), **8**, and **14**, as well as substituted benzene substrates e.g., toluene and chlorobenzene **17**, all revealed evidence of edge-to-face (T-bonding) interactions with Phe-216 and His-222. The simultaneous edge-to-face interactions, of amino acid residues Phe-216 and His-222, with both the phenyl and pyridyl rings of substrates **1**, **8**, and **14** in the present context, were noteworthy. Docking distances between the proximate T-bonding H atoms and arene faces of quinoline **1** (Supplementary Figures 4A_1_-A_3_) were estimated to be within the range 2.6–2.8 Å, assuming a C-H bond length of 1.08 Å. These inter-ring distances are in accord with the calculated interacting H-to-ring center perpendicular distances, of *ca*. 2.6–2.8 Å and observed distances *ca*. 2.70–2.86 Å, from X-ray crystallographic analysis, using a range of model systems (Hoering et al., 2016; Boyd et al., 2019). Keyhole pictures, looking through the rear face of the component aromatic ring, showed both edge to face-T (Supplementary Figures 4A_3_,B_3_,C_3_), and face tilted-T dockings (Supplementary Figures 4A_2_,B_2_,C_2_) of quinoline **1** with TDO. This was consistent with the attractive interactions between phenyl rings (Supplementary Figures 4A_1_,B_1_), phenyl/pyridyl rings (Supplementary Figure 4C_1_), phenyl/imidazoyl rings (Supplementary Figures 4A_1_,B_1_), and pyridyl/imidazoyl rings (Supplementary Figure 4C_1_).

It was concluded that the observed absolute configurations of the isolated carbocyclic ring *cis*-dihydrodiol metabolites **2**, **3**, **9**, and **10** were in agreement with preferred *in silico* docking orientations of the corresponding substrates (Figures 4A,B, 5A,B). The preferred orientations of quinoline **1** (Figure 4C), 2-chloroquinoline **8** (Figure 5C), and 2-chloropyridine **14** (Figure 6), would also be supportive of TDO-catalyzed *cis*-dihydroxylation occurring at the pyridine rings to give the undetected *cis*-dihydrodiol intermediates **7**, **12**, and **15**.

### Chemoenzymatic Synthesis of Isolated and Potential Arene Oxide Metabolites of Quinoline

The docking studies described in Figures 4A,B, 5A,B enabled us to develop novel chemoenzymatic approaches for the preparation of different types of chiral azaarene derivatives. The availability of the *cis*-diol **10** as the major metabolite from TDO-catalyzed *cis*-dihydroxylation of 2-chloroquinoline **8**, resulted in its first use in chemoenzymatic synthesis, when chiral 2,2′-bipyridines and 2,2′-bipyridine *N*-oxides were produced (Boyd et al., 2008, 2010). These compounds were found to be useful ligands for asymmetric oxidation (→ 97% ee) (Boyd et al., 2008), cyclopropanation (→ 95% ee) (Boyd et al., 2008), aminolysis (→ 84% ee) (Boyd et al., 2010), and allylation reactions (→ 86% ee) (Boyd et al., 2010). Further possible applications of both *cis*-diol metabolites **9** and **10**, to the chemoenzymatic synthesis of detected and possible mammalian metabolites of quinolines, were also developed.

The identification of a 1,2-epoxide, a mammalian metabolite of naphthalene (Jerina et al., 1968), resulting from cytochrome P-450 (CYP-450)-catalyzed epoxidation, led to a quest for other examples of arene oxide metabolites. A similar type of monooxygenase-catalyzed dearomatization was later found to yield epoxide metabolites of both monocyclic arenes (e.g., benzene 1,2-oxide) and polycyclic azaarenes (e.g., quinoline 5,6-oxide) (Agarwal et al., 1986). Arene dioxides, *trans*-dihydrodiols, *trans*-tetrahydrodiol epoxides, and tetrahydroarene tetrols were later identified as other mammalian metabolites of naphthalene (Stillwell et al., 1978, 1982; Horning et al., 1980). Further studies established that liver metabolism of larger (tetracyclic and pentacyclic) bay-region arene and heteroarene substrates often involved epoxide intermediates associated with their mutagenicity and tumorigenicity. The metabolic sequence: arenes → arene oxides → *trans*-dihydrodiols → *trans*-tetrahydrodiol epoxides → DNA-adducts, became known as the *Bay-region Mechanism* (Buening et al., 1978; Karle et al., 2004; Chang et al., 2013).

**Figure 8:**
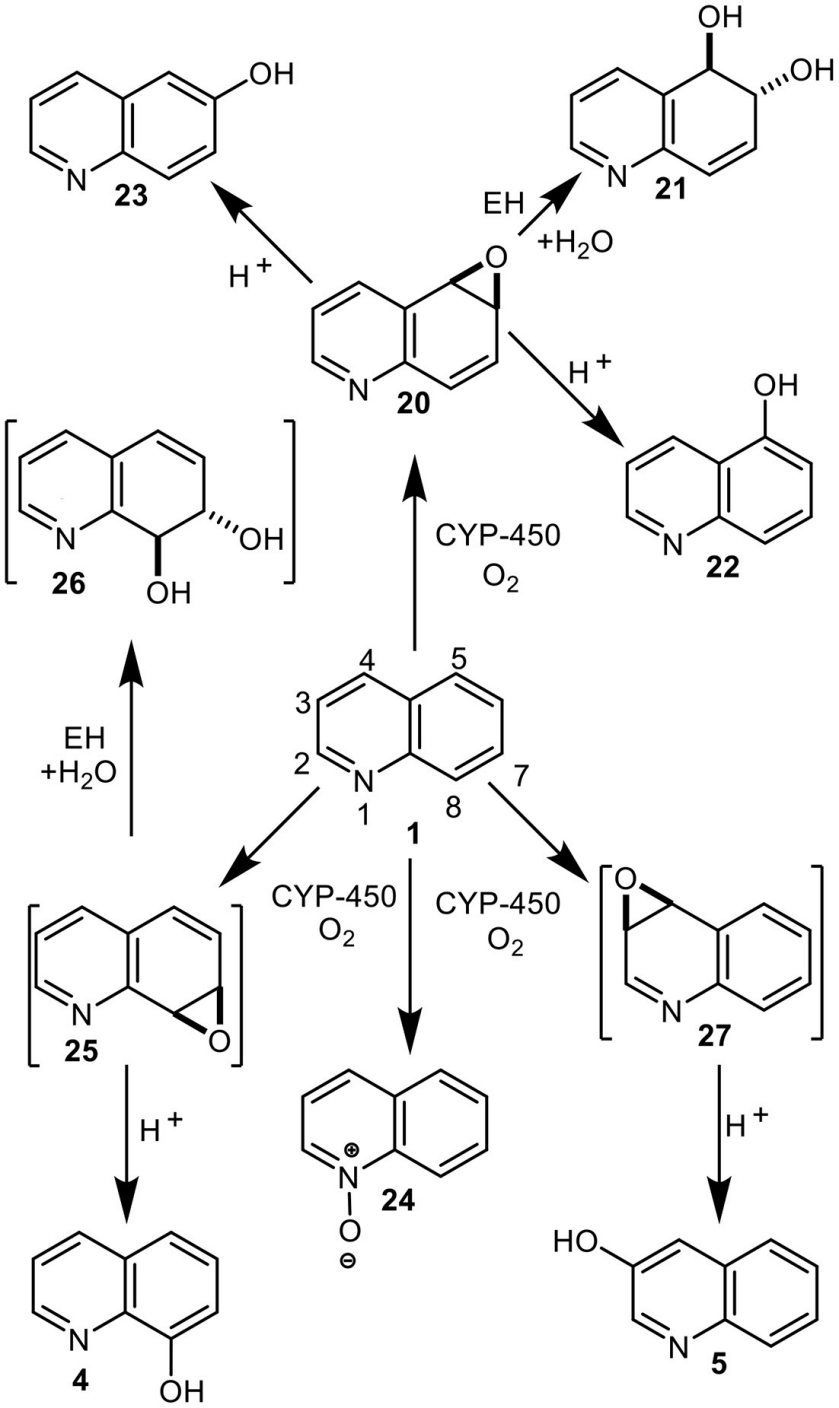
Mammalian metabolites of quinoline **1**, both isolated (**4**, **5**, **20–24**) and postulated (**25–27**).

Identification of mammalian metabolites of quinoline **1** and their role in its mutagenicity and tumorigenicity, has been of interest for more than 40 years (Hollstein et al., 1978; Tada et al., 1980, 1982; LaVoie et al., 1983; Agarwal et al., 1986, 1990; Willems et al., 1992; Saeki et al., 1993; Reigh et al., 1996; Suzuki et al., 2000; Dowers et al., 2004; Hakura et al., 2007; Diaz Duran et al., 2015; Matsumoto et al., 2018). Several quinoline arene oxides, e.g., **20** and **27**, were initially postulated as possible liver microsomal metabolites of quinoline **1** (Hollstein et al., 1978; Tada et al., 1980, 1982; LaVoie et al., 1983), before 5,6-arene oxide **20** was finally confirmed as a major metabolite (Figure 8) (Agarwal et al., 1986). Epoxide hydrolyase-catalyzed hydrolysis of the identified 5,6-arene oxide **20** would also account for the isolated *trans*-dihydrodiol metabolite **21**, and aromatization of the undetected 3,4-arene oxide **27** could yield the isolated 3-hydroxyquinoline **5**. Transient arene oxides, including compound **27**, resulting from CYP-450-catalyzed epoxidation of quinoline **1**, were initially postulated to be responsible for its mutagenicity and carcinogenicity (Hollstein et al., 1978; Tada et al., 1980, 1982; LaVoie et al., 1983). Liver microsomal metabolism of quinoline **1** also yielded DNA adducts, which on hydrolysis, under acidic or basic conditions, gave 3-hydroxyquinoline **5**. Efforts to identify mammalian metabolic pathways responsible for the mutagenicity (and carcinogenicity) of quinoline **1** (Willems et al., 1992; Suzuki et al., 2000), required the chemical synthesis of identified, and possible, metabolites of similar structures to those derived earlier from naphthalene (e.g., phenols, arene oxides, arene dioxides, *trans*-dihydrodiols, and *trans-*tetrahydrodiol epoxides) (Jerina et al., 1968; Stillwell et al., 1978, 1982; Horning et al., 1980).

The syntheses of racemic quinoline arene oxides (**20**, **25**), *trans*-dihydrodiols (**21**, **26**), *syn*- and *anti*- arene dioxides (**28** and **29**), and *trans*-diol epoxides (**30** and **31**), were previously reported (Agarwal et al., 1986; Boyd et al., 1989, 1994; Bushman et al., 1989; Willems et al., 1992) (Figures 8, **10**). These chiral compounds, along with achiral metabolites including *N*-oxide **24** and phenols **4**, **5**, **22**, and **23**, were tested for mutagenicity, using the Ames/*Salmonella* microsomes method (Willems et al., 1992; Suzuki et al., 2000). As none of these metabolites or possible metabolites, were found to be more mutagenic than quinoline **1**, it was assumed that a metabolic sequence similar to that in the *Bay-region Mechanism*, involving racemic arene oxides, e.g., **20**→*trans*-dihydrodiols **21**→*trans*-tetrahydrodiol epoxides **31**, was unlikely to be involved in its mutagenicity. It was later established that the particular absolute configurations of polycyclic arene metabolites, associated with the *Bay-region Mechanism*, had a marked effect on their carcinogenicity (Buening et al., 1978; Karle et al., 2004; Chang et al., 2013). The mutagenicity of the enantiopure derivatives of quinoline **20**, **21**, **25**, **26**, **28**, **29**, **30**, and **31** was not investigated.

**Figure 9:**
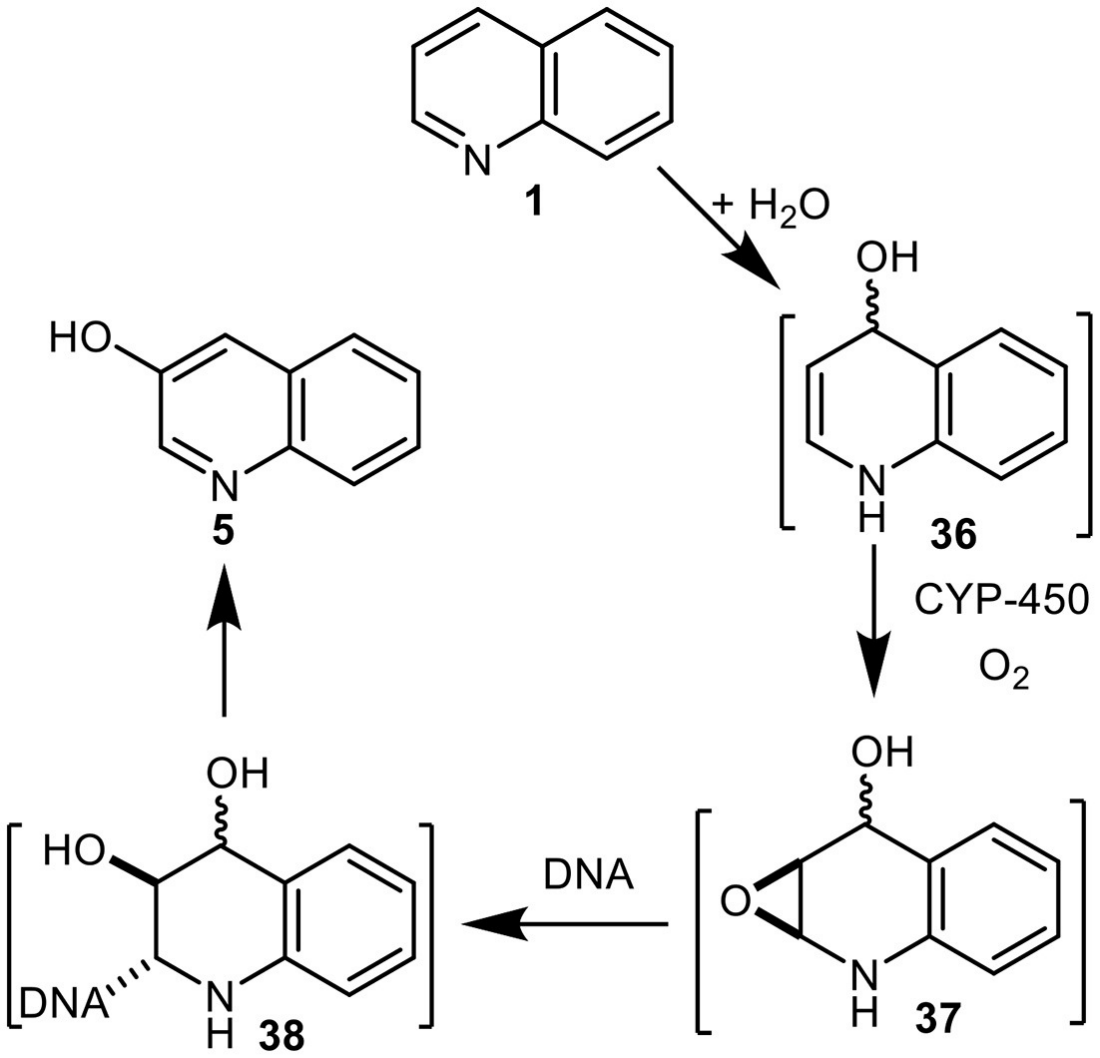
Proposed metabolic sequence for quinoline **1** involving intermediates **36–38**.

An alternative metabolic pathway for quinoline **1** in mammals was proposed involving dearomatization of the pyridyl ring and formation of the undetected quinoline hydrate **36** (Saeki et al., 1993, 1996, 1997a,b; Suzuki et al., 2000) (Figure 9). While relatively stable arene hydrate derivatives of quinoline were isolated earlier as metabolites of 5,6- and 7,8-dihydroquinolines (Agarwal et al., 1994), compound **36**, being both an enamine and an arene hydrate, would be expected to be less stable. The CYP-450-catalyzed epoxidation of enamine intermediate **36** was postulated to give the undetected epoxide **37** and to react with DNA to yield unstable adduct **38**, which rearomatized under both acidic and basic conditions to yield 3-hydroxyquinoline **5** (Saeki et al., 1993, 1996, 1997a,b; Suzuki et al., 2000). The metabolic pathway **1**→**36**→**37**→**38** (*Enamine Epoxide Theory*, Figure 9) was thus proposed to account for the mutagenicity and carcinogenicity of quinoline **1**. It is noteworthy that while some substituted quinolines were found to be carcinogenic, 2-chloroquinoline **8** was not (Hirao et al., 1976; Hakura et al., 2007).

**Figure 10:**
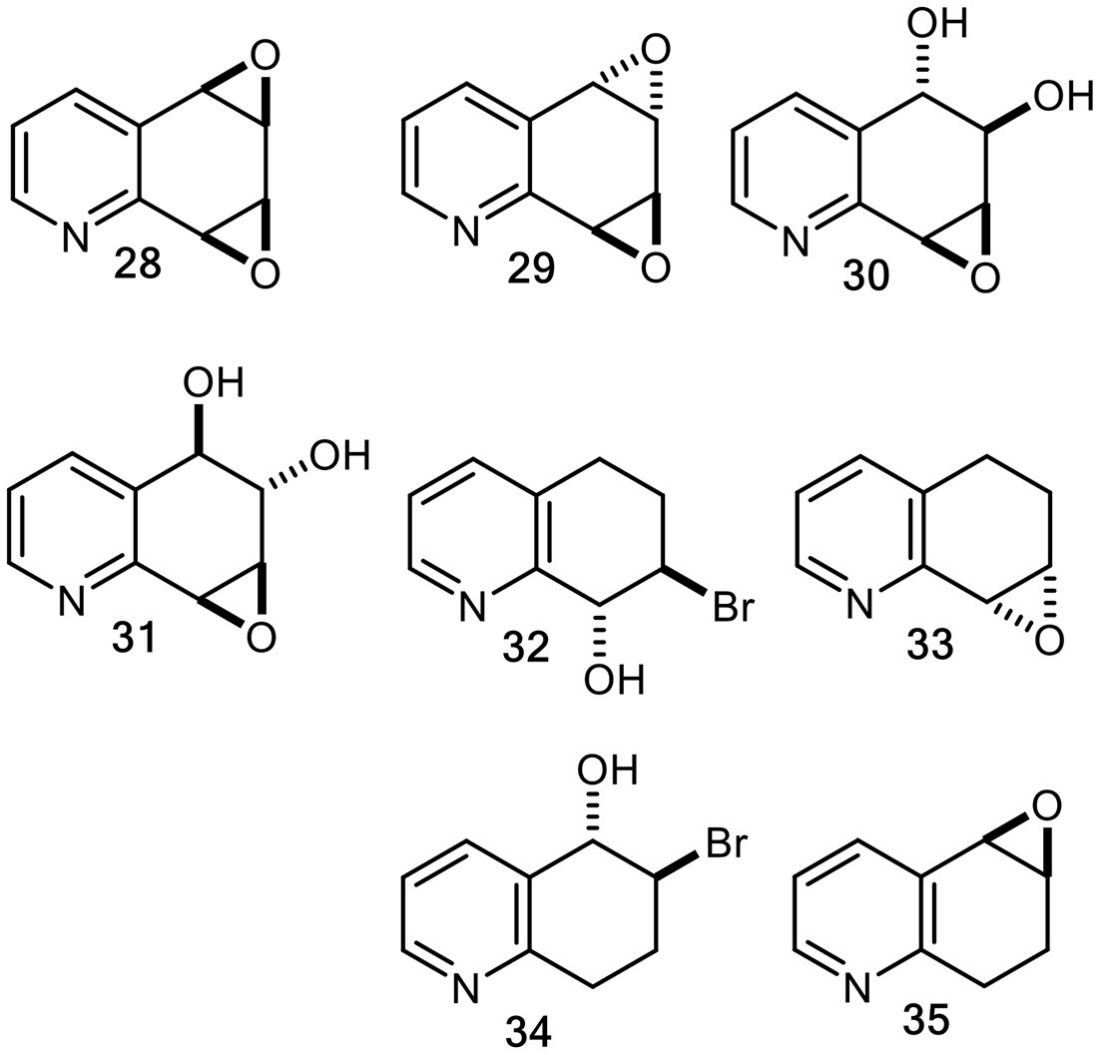
Chemically synthesized racemic quinoline *syn*- and *anti*-dioxides (**28** and **29**), diol epoxides (**30** and **31**), tetrahydrobromohydrins (**32** and **34**), and derived tetrahydroepoxides (**33** and **35**).

In the context of mutagenesis studies of known and possible mammalian metabolites of quinoline **1** (Willems et al., 1992), our multistep chemical synthesis routes to racemic quinoline oxides (**20** and **25**) and quinoline dioxides (**28** and **29**), were achieved *via* tetrahydrobromohydrins (**32**, **34**) and tetrahydroepoxides (**33**, **35**) intermediates (Figure 10) (Agarwal et al., 1986, 1990; Boyd et al., 1989, 1991b).

While the individual enantiomers of arene oxides **20** and **25** were separable by analytical chiral stationary phase (CSP) HPLC, this analytical scale approach was unsuitable for further chemoenzymatic synthesis studies and therefore alternative preparative methods for obtaining these enantiopure compounds were developed. The chemical resolution of bromohydrin derivatives of 1,2,3,4-polycyclic tetrahydroarenes as MTPA esters provided a generally applicable route to a range of enantiopure arene oxide derivatives, e.g., naphthalene 1,2-oxide and anthracene 1,2-oxide (Akhtar et al., 1979). Further application of this MTPA ester resolution method to racemic bromohydrin **32**, yielded enantiopure tetrahydroepoxides (7*S*,8*R*)-**33** ([α]_D_ +157) and (7*R*,8*S*,)-**33** ([α]_D_ −157) intermediates and finally quinoline 7,8-oxide, (7*S*,8*R*)-**25** ([α]_D_ +55) and (7*R*,8*S*)-**25** ([α]_D_ −55, unpublished data). The racemic tetrahydroquinoline bromohydrin **34** was also resolved, but as dibenzotartrate salts to give enantiomers of quinoline 5,6-oxide (5*R*,6*S*)-**20** ([α]_D_ −23) and (5*S*,6*R*)-**20** ([α]_D_ +23) *via* tetrahydroepoxides (5*R*,6*S*)-**35** and (5*S*,6*R*)-**35** (Akhtar et al., 1979). The enantiopurities (>98% *ee*) of arene oxides **20** and **25** were confirmed by CSPHPLC analysis; more than ten synthetic steps from quinoline **1** were required using these chemical resolution methods.

**Figure 11:**
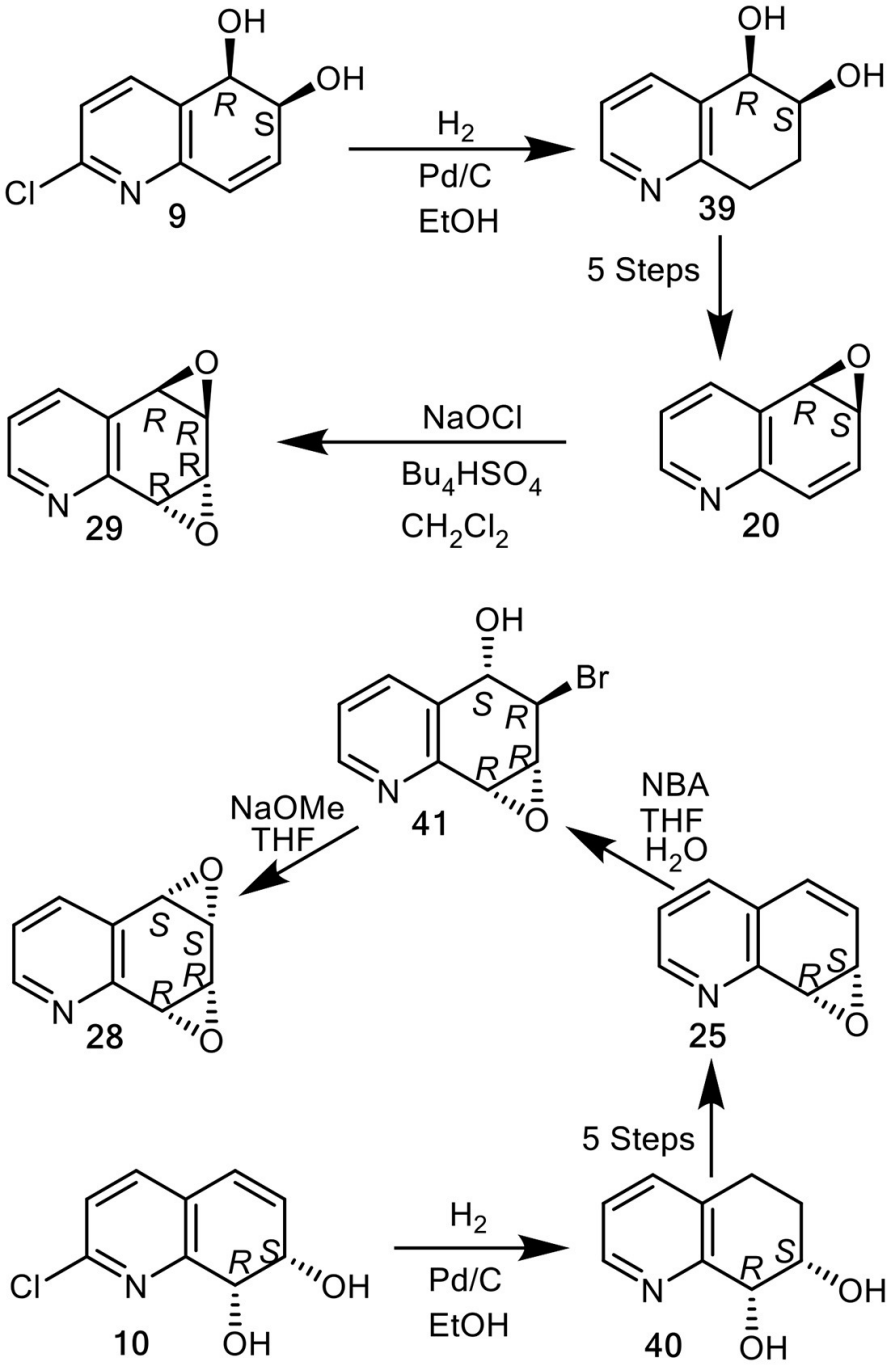
Synthesis of quinoline arene oxides (–)-**20**, (+)-**25**, and arene dioxides (+)-*syn*-**28** and (–)-*anti*-**29** from *cis*-dihydrodiols (+)-**10** and (**+**)**-9**.

An alternative chemoenzymatic approach to the synthesis of arene oxide enantiomers **20** and **25**, using the quinoline *cis*-dihydrodiol metabolites (5*R*,6*S*)-**2** and (7*S*,8*R*)-**3** (X = H) as precursors was then developed (Boyd et al., 1991a). This again produced the quinoline oxide enantiomers (–)-(5*R*,6*S*)-**20** and (+)-(7*S*,8*R*)-**25**, in seven steps from quinoline **1**, *via* tetrahydroepoxides (5*R*,6*S*)-**35** and (7*S*,8*R*)-**33**. Although this chemoenzymatic approach required fewer steps than the earlier methods involving the chemical resolution of bromohydrins **32** and **34** (Boyd et al., 1994), the very low (<5%) isolated yields of the less stable *cis*-dihydrodiol precursors **2** and **3** obtained by *P. putida* UV biotransformation (Boyd et al., 1993), was a limiting factor. As the isolated yields of the more stable *cis*-dihydrodiol metabolites **9** (9%) and **10** (21%), obtained from of 2-chloroquinoline **8** were higher, under similar conditions, they were used as alternative starting compounds to yield *cis*-tetrahydrodiol intermediates (5*R*,6*S*)-**39** and (7*S*,8*R*)-**40** in the current study (Figure 11).

(5*R*,6*S*)-*cis*-Tetrahydrodiol **39** ([α_D_] −7, CHCl_3_) and (7*S*,8*R*)-*cis*-tetrahydrodiol **40** ([α_D_] −72, CHCl_3_), known precursors of arene oxides **20** and **25** (Boyd et al., 1994) were efficiently synthesized (*ca*. 80% yield), by catalytic hydrogenation/hydrogenolysis (H_2_, Pd/C, EtOH) of the (5*R*,6*S*)-*cis*-dihydrodiol **9** ([α_D_] +140, MeOH) and (7*S*,8*R*)-*cis*-dihydrodiol **10** ([α_D_] +148, MeOH), respectively. This new approach avoided use of the less stable *cis*-dihydrodiol metabolites **2** and **3** and provided an improved chemoenzymatic route the corresponding arene oxides (–)-**20** and (+)-**25** (Figure 11) (Boyd et al., 1994).

Attempted epoxidation of available arene oxides **20** and **25** to yield the arene dioxide isomers **28** and **29**, using several oxidants, including *m*-chloroperoxybenzoic acid and dimethyl dioxirane, resulted in the formation of *N*-oxides. The epoxidation of arene oxides **20** and **25** in CH_2_Cl_2_ solution was achieved using sodium hypochlorite solution (pH 8.6) in the presence of tetrabutylammonium hydrogen sulfate and gave *anti*-quinoline dioxide **29** exclusively. With arene oxide (–)-**20** available from earlier studies (Boyd et al., 1991a, 1994), NaOCl oxidation gave the (5*R*,6*R*,7*R*,8*R*)—arene dioxide **29** ([α]_D_ −100, 38% yield, Figure 11). Reaction of the (+)-(7*S*,8*R*) enantiomer of arene oxide **25** with *N*-bromoacetamide under reported conditions, yielded the crude *trans*-bromohydrin intermediate **41**, which upon treatment with sodium methoxide furnished the *syn*-quinoline dioxide (5*S*,6*S*,7*R*,8*R*)-**28** ([α]_D_ +25, Figure 11) (Boyd et al., 1991b).

A further approach to the synthesis of the *syn*-quinoline dioxide enantiomers (–) and (+)-**28** involved the *cis*-dihydroxylation of the 5,6-(+)-**9** and 7,8 *cis*-dihydrodiol (+)-**10**. Catalytic *cis*-hydroxylation (OsO_4_, Me_3_NO) of 2-chloroquinoline *cis*-dihydrodiol (+)-**9**, produced an isomeric mixture of polar tetrols **42** and **43** that could not be separated by chromatography and were converted directly to the corresponding tetraacetate derivatives **44** and**45** (1:3, Figure 12). Purification of the crude tetraacetates was achieved by flash chromatography followed by multiple elution PLC.

**Figure 12:**
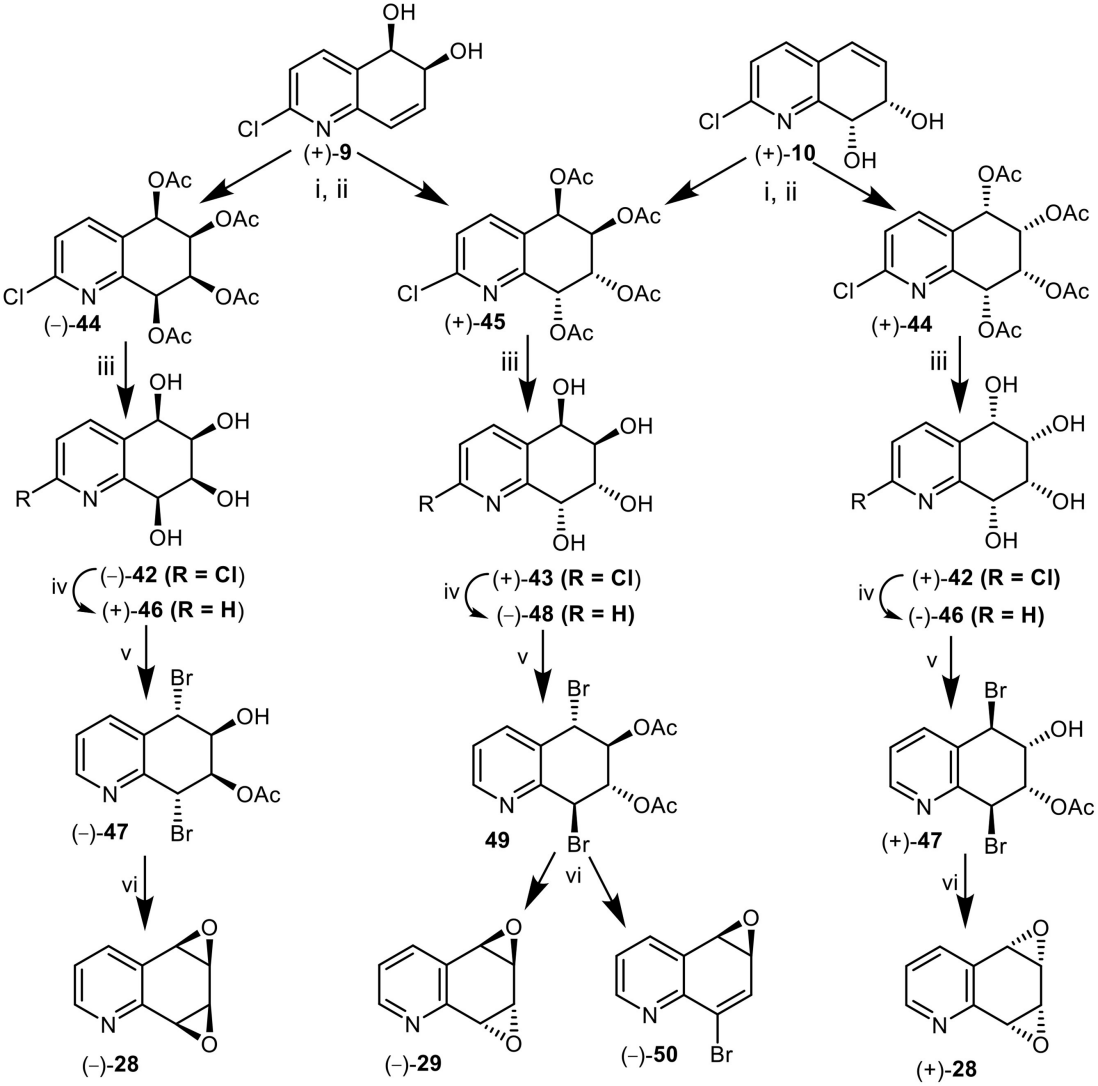
Synthesis of *anti* (–)-**29** and *syn*-quinoline dioxides (–)-**28** and (+)-**28** from *cis*-dihydrodiols (+)-**9** and **(+)-10**. Reagents: (i) OsO_4_, Me_3_NO-CH_2_Cl_2_; (ii) Ac_2_O-pyridine; (iii) NH_3_, MeOH; (iv) H_2_, Pd-C, MeOH; (v) AcOMe_2_COBr, MeCN; (vi) NaOMe, Et_2_O.

Hydrolysis of the minor *syn*-tetraacetate enantiomer (–)-**44**, under basic conditions (NH_3_, MeOH) yielded the 2-chloroquinoline *syn*-tetrol (–)-**42** which was converted to the corresponding quinoline tetrol (+)-**46**, by hydrogenolysis (Pd-C). Treatment of tetrol (+)-**46** with acetoxyisobutrylbromide in MeCN solution yielded mainly a dibromoacetate, tentatively assigned as (–)-**47**; it was separated from trace amounts of the expected dibromodiacetate by PLC. Dibromoacetate **47** was formed by partial hydrolysis of the dibromodiacetate during the isolation process. Treatment of dibromomonoacetate (–)-**47** with NaOMe in THF yielded the *syn*-arene dioxide enantiomer (–)-**28** from 5,6-*cis*-dihydrodiol (+)-**9** in the sequence (+)-**9** → (–)-**44** → (–)-**42** → (+)-**46** → (–)**-47** → (–)-**28 (****Figure 12****)**.

In a similar manner metabolite (+)-**10** was converted into the mixture of tetraacetates **44** and **45** (1:3). The minor *syn*-tetraacetate (+)-**44**, was separated by PLC; it was assigned the opposite (5*S*,6*S*,7*R*,8*R*) absolute configuration. The *syn-*tetraacetate (+)-**44** was used in an enantiocomplementary synthetic sequence (+)-**10** → (+)-**44** → (+)-**42** → (–)-**46** → (+)-**47** → (+)-**28 (****Figure 12****)**.

*anti*-Tetraacetate (5*R*,6*R*,7*R*,8*R*) enantiomer (+)-**45** obtained from 5,6-(+)-**9** and 7,8-(+)-**10** *cis*-dihydrodiol metabolites, was treated in a similar manner to the *syn*-tetraacetate enantiomers (+)-**44** and (–)-**44** to yield tetrols (+)-**43**, (–)-**48**, and dibromodiacetate -**49** (Figure 12). Treatment of bromoacetate **49** with sodium methoxide yields a mixture of the expected *anti*-quinoline dioxide (–)-**29** as the major product accompanied by a minor compound which was identified as 8-bromoquinoline-5,6-oxide **50**. A competing E1cB mechanism could account for the unexpected loss of acetic acid to form the minor product, arene oxide **50**. These products were thus formed *via* the synthetic sequence (+)-**9** or (+)-**10** → (+)-**45** → (+)-**43** → (–)-**48**→**49** → (–)-**29** + (–)-**50** (Figure 12).

## Discussion

Both Autodock and Gold programs proved to be very successful in matching the preferred *in silico* orientations of arene substrates with the experimentally confirmed regiochemistry and absolute stereochemistry of the isolated *cis*-diol metabolites. Previous docking studies of arene substrates with TDO indicated that the preferred orientations were controlled by: (i) attractive edge-to-face T shaped interactions with the orthogonal phenyl group of Phe-216 and imidazole ring of His 222 and (ii) Van der Waals interactions with the proximate hydrophobic amino acids Ile-276, Leu-272, Ile-324, Val-309, Leu-272, Phe-352 (Hoering et al., 2016; Vila et al., 2016a,b, 2017; Boyd et al., 2017, 2019). Theoretical, crystallographic and experimental support for phenyl-phenyl and phenyl-pyridyl edge-to-face bonding (T-bonding) interactions, between arene and heteroarene rings, has been reported (Jennings et al., 2001; Escudero et al., 2009; Gonzalez-Rosende et al., 2017; Aliev and Motherwell, 2019). Little evidence was available, from crystalline protein structures, for similar edge-to-face interactions between the imidazole ring of histidine with other aromatic residues, e.g., Phe, Tyr, Trp, His (Bhattacharyya et al., 2003). The possibility of further types of edge-to-face interactions between heteroarene substrates **1** (Figures 4A–C), **8** (Figures 5A–C), and **14** (Figure 6) with Phe-216 and His-222 in the active site of TDO (Figures 4–6), is also apparent.

Based on the docking orientations of 2-chloroquinoline **8** (Figures 5A,C), *cis*-dihydroxylation could also occur at the 3,4-bond of the pyridine ring. The shorter distance between this bond and dioxygen in Figure 5C (3.3–3.4 Å), would appear to favor formation of the undetected (3*R*,4*R*)-dihydrodiol **12**, over the (3*S*,4*S*) configuration resulting from the orientation of substrate **8** shown in Figure 5A (3.6–4.0 Å). Conversely, the higher binding energy, −5.8 kcal/mol^−1^, associated with Figure 5A relative to Figure 5C (−5.1 kcal/mol^−1^), would suggest a preference for the (3*S*,4*S*) absolute configuration, thus making a prediction of the preferred stereochemistry of *cis*-dihydrodiol **11** less reliable.

To account for the unexpected isolation of (3*S*,4*S*)-diol **11** as a product from *P. putida* UV4 biotransformations of 2-chloroquinoline **8**, the undetected *cis*-dihydrododiol **12** of unknown absolute configuration, was originally proposed as a possible intermediate in the metabolic sequence **8**→**12**→**11** (Figure 2) (Boyd et al., 1998). Later GC-MS identification of 2-quinolone **13** as a very minor metabolite of substrate **8**, was consistent with an alternative or additional metabolic pathway (**8**→**13**→**11**, Figure 2) (Boyd et al., 2002). Stronger evidence for the latter biosynthetic route was provided when 2-quinolone **13** was used as substrate and (3*S*,4*S*)-diol **11** was found to be the only identifiable metabolite. Since 2-hydroxyquinoline is a minor tautomer of 2-quinolone **13**, the formation of *cis*-diol metabolite **11** could also be considered as a TDO-catalyzed *cis*-dihydroxylation within a substituted pyridyl ring. It is therefore possible that *cis*-diol metabolite **11** was formed from 2-chloroquinoline **8**, *via* both 2-quinolone **13** and *cis*-dihydrodiol **12** intermediates.

The previously reported regioselective and enantioselective *cis*-dihydroxylation, of the carbocyclic ring of quinoline **1**, 2-chloroquinoline **8** and 2-chloropyridine **14**, was rationalized by molecular docking (Autodock Vina) studies of these substrates. The *in silico* results revealed novel edge-to-face attractive interactions at the active site of TDO. These studies also provided support for the TDO-catalyzed *cis*-dihydroxylation of: (i) the pyridyl ring in substrates, **1**, **8**, and **14**, to yield the corresponding unstable *cis*-dihydrodiol metabolites, **7**, **12**, and **15**, and (ii) the carbocyclic ring to give stable *cis*-dihydrodiol metabolites **2**, **3**, **9**, and **10** having the correct absolute configurations. Evidence for the monooxygenase-catalyzed epoxidation and dioxygenase-catalyzed *cis*-dihydroxylation of a pyridyl ring was discussed, in the context of both bacterial and mammalian metabolism of quinoline **1**.

Our findings are consistent with the current thinking on the catalytic mechanism for these enzymes, which is not fully understood. Barry and Challis (2013) discuss an important point, i.e., whether the Fe(III)-OOH complex in the TDO catalytic cycle reacts directly with the substrate or first undergoes rearrangement to an Fe(V)-O(OH) complex. They highlight the question of which arene carbon atom first forms a bond with an oxygen atom.

In conclusion, new synthetic applications of the relatively stable *cis*-diol metabolites **9** and **10**, derived from 2-chloroquinoline **8** were successfully investigated. These *cis*-dihydrodiols were utilized in the chemoenzymatic synthesis of the corresponding *cis*-tetrahydrodiols **39** and **40**, required in the synthesis of enantiopure quinoline arene oxides, (–)-**20** and (+)-**25**. This process was greatly facilitated by molecular docking preliminary studies. The unexpected 8-bromoquinoline oxide (–)-**50** was obtained during the synthesis of *anti*-(–)-**29** and *syn*-quinoline dioxide enantiomers (–)-**28** and (+)-**28** from *cis*-dihydrodiols **9** and **10**.

## Data Availability Statement

The datasets presented in this study can be found in online repositories. The names of the repository/repositories and accession number(s) can be found in the article/Supplementary Material.

## Author Contributions

CA, DB, NS, and PS supervised the research, designed the research, obtained the funding, wrote the manuscript, and designed the experiments. PH conducted the modeling and docking. JC, PL, and NS conducted the laboratory synthesis/biotransformations. All authors contributed to the article and approved the submitted version.

## Conflict of Interest

The authors declare that the research was conducted in the absence of any commercial or financial relationships that could be construed as a potential conflict of interest.
