# Supplementary material for: Toluene Dioxygenase-Catalyzed cis-Dihydroxylation of Quinolines: A Molecular Docking Study and Chemoenzymatic Synthesis of Quinoline Arene Oxides
Source: Front Bioeng Biotechnol. 2021 Feb 12;8:619175. doi: 10.3389/fbioe.2020.619175 (PMC7907597; doi:10.3389/fbioe.2020.619175)
Supplement: Supplementary file 1 [file Data_Sheet_1.docx]

Toluene Dioxygenase-Catalysed *cis*-Dihydroxylation of Quinolines: A Molecular Docking and Chemoenzymatic Synthesis Study of Quinoline Arene Oxides

Derek R. Boyd,^a*^ Narain D. Sharma,^a^ Pui L. Loke,^a^ Jonathan G. Carroll,^a^ Paul J. Stevenson, ^a^ Patrick Hoering^b^ and Christopher C. R. Allen^b*^

*^a^ School of Chemistry and Chemical Engineering, Queen’s University of Belfast, Belfast, BT9 5AG, UK E-mail:* [*dr.boyd@qub.ac.uk; Tel:+44*](about:blank) *(0) 289097442*

*^b^ School of Biological Sciences, Queen’s University of Belfast, Belfast, BT9 5AJ, UK*

**Supplementary Information**

**Quinoline, 2-chloroquinoline, 2-chloropyridine and chlorobenzene dockings**

S-3: Figures SI: 4D, SI: 5D, SI: 6, SI: 7

S-4: Figures SI: 4A_1_, SI: 4A_2_, SI: 4A_3_, SI:4B_1_, SI: 4B_2_, SI: 4B_3_.

S-5: Figures SI: 4C_1_, SI: 4C_2_, SI: 4C_3_.

**Proton NMR spectra**

S-6: (5*R*,6*R*,7*R*,8*R*)-5,6,7,8-Tetraacetoxy-2-chloro-5,6,7,8-tetrahydroquinoline (**42**)

S-7: (5*S*,6*S*,7*R*,8*R*)-5,6,7,8-Tetraacetoxy-2-chloro-5,6,7,8-tetrahydroquinoline (**43**)

S-8: (5*S*,6*S*,7*R*,8*R*)-2-chloro-5,6,7,8-tetrahydroquinoline-5,6,7,8-tetrol (**44**)

S-9: (5*S,*6*S,*7*R,*8*R)-5,*6,7,8-Tetrahydroquinoline-5,6,7,8-tetrol (**45**)

S-10: (5*R*,6*R*,7*S*,8*S*)-5,8-Dibromo-6-hydroxy-5,6,7,8-tetrahydro-7-quinolinyl acetate (**46**)

**ECD spectra**

S-11: (+)-(5*S*,6*S*,7*R*,8*R*)- Quinoline-5,6,7,8-dioxide (**34**)

S-12: (-)-(5*R*,6*R*,7*S*,8*S*)- Quinoline-5,6,7,8-dioxide **(34**)

**Toluene dockings**

S-13: Figures SI: A, B, C, D

**SI: 4D SI: 5D**


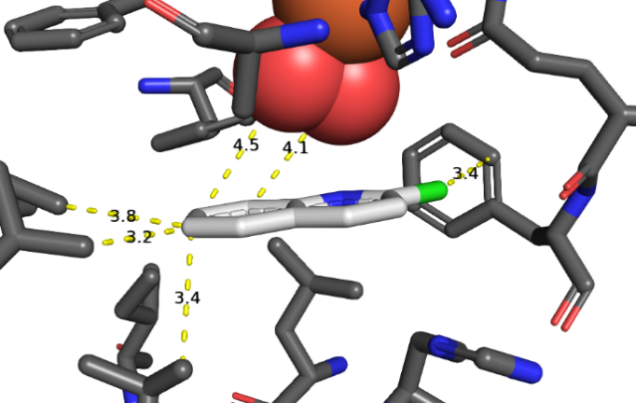

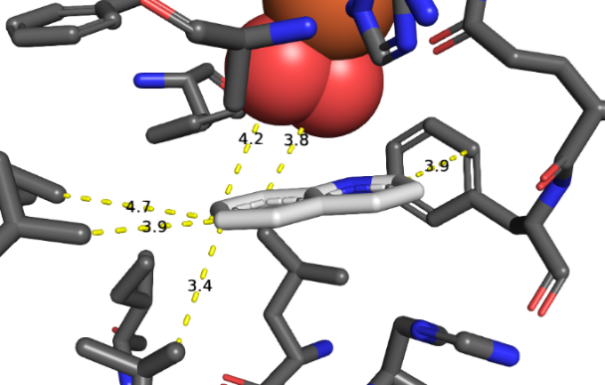


**7,8-attack (-5.8 kcal.mol^-1^) 7,8-attack (-6.2 kcal.mol^-1^)**

**7,8-(3.8-4.2 Å) 7,8-(4.1-4.5 Å)**


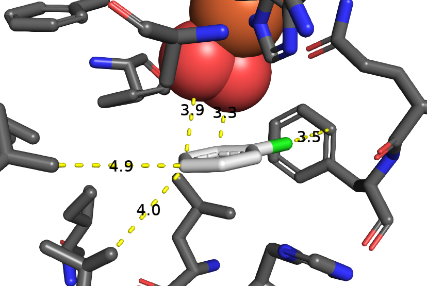
 **SI: 6 SI: 7**


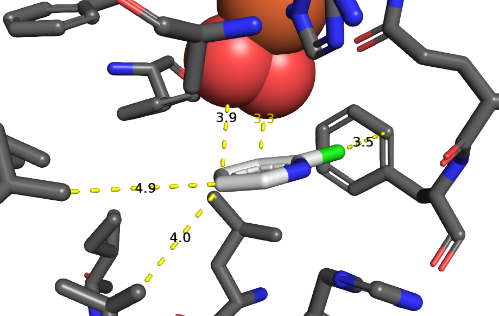


**3,4-attack (-5.0 kcal.mol^-1^) 2,3-attack (-5.1 kcal.mol^-1^)**

**3,4-(3.3-3.9 Å) 2,3-(3.3-3.9 Å)**

**Figures. TDO docking of quinoline 1, (SI: 4D), 2-chloroquinoline 8, (SI: 5D), 2-chloropyridine 1 (SI: 6), and of chlorobenzene 17 (SI: 7).**

**SI: 4A_1_ SI: 4A_2_ SI: 4A_3_**


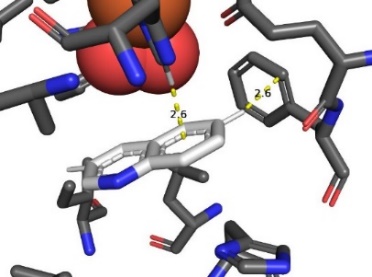

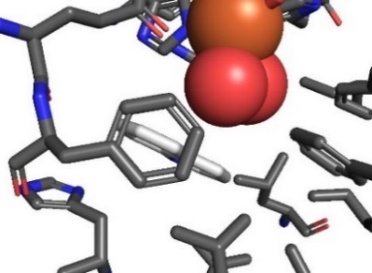

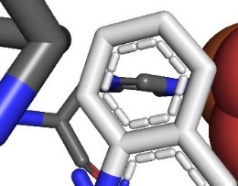


**Phe216-phenyl-His228 Phe216-phenyl Phenyl-His228**

**Figures SI: 4A_1_, SI: 4A_2_, SI: 4A_3_. Preferred orientations for *cis*-dihydroxylation of quinoline 1 at the 5,6-bond**

**SI: 4B_1_ SI: 4B_2_ SI: 4B_3_**


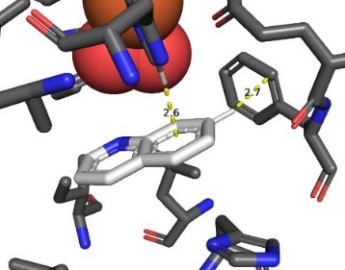

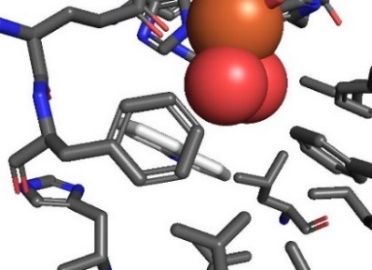

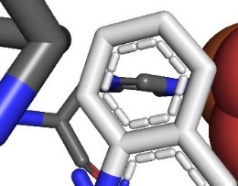


**Phe216-phenyl-His228 Phe216-phenyl Phenyl-His228**

**Figures SI: 4B_1_, SI: 4B_2_, SI: 4B_3_.** **Preferred orientation for *cis*-dihydroxylation of quinoline 1 at the 7,8-bond**

**SI: 4C_1_ SI: 4C_2_ SI: 4C_3_**


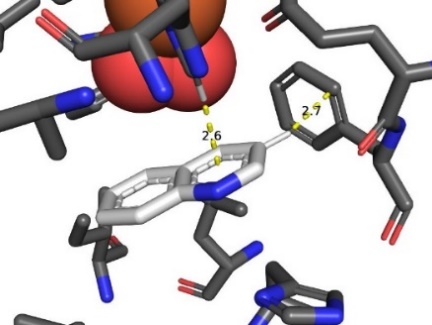

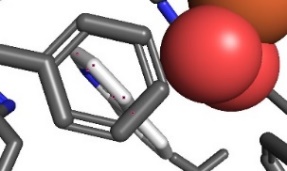

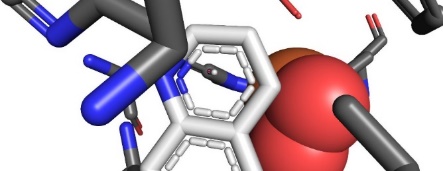


**Phe216-pyridyl- His228 Pyridyl- His228 Phe216-pyridyl**

**Figures SI: 4C_1_, SI: 4C_2_, SI: 4C_3_.** **Preferred orientation for *cis*-dihydroxylation of quinoline 1 at the 3,4-bond.**

**
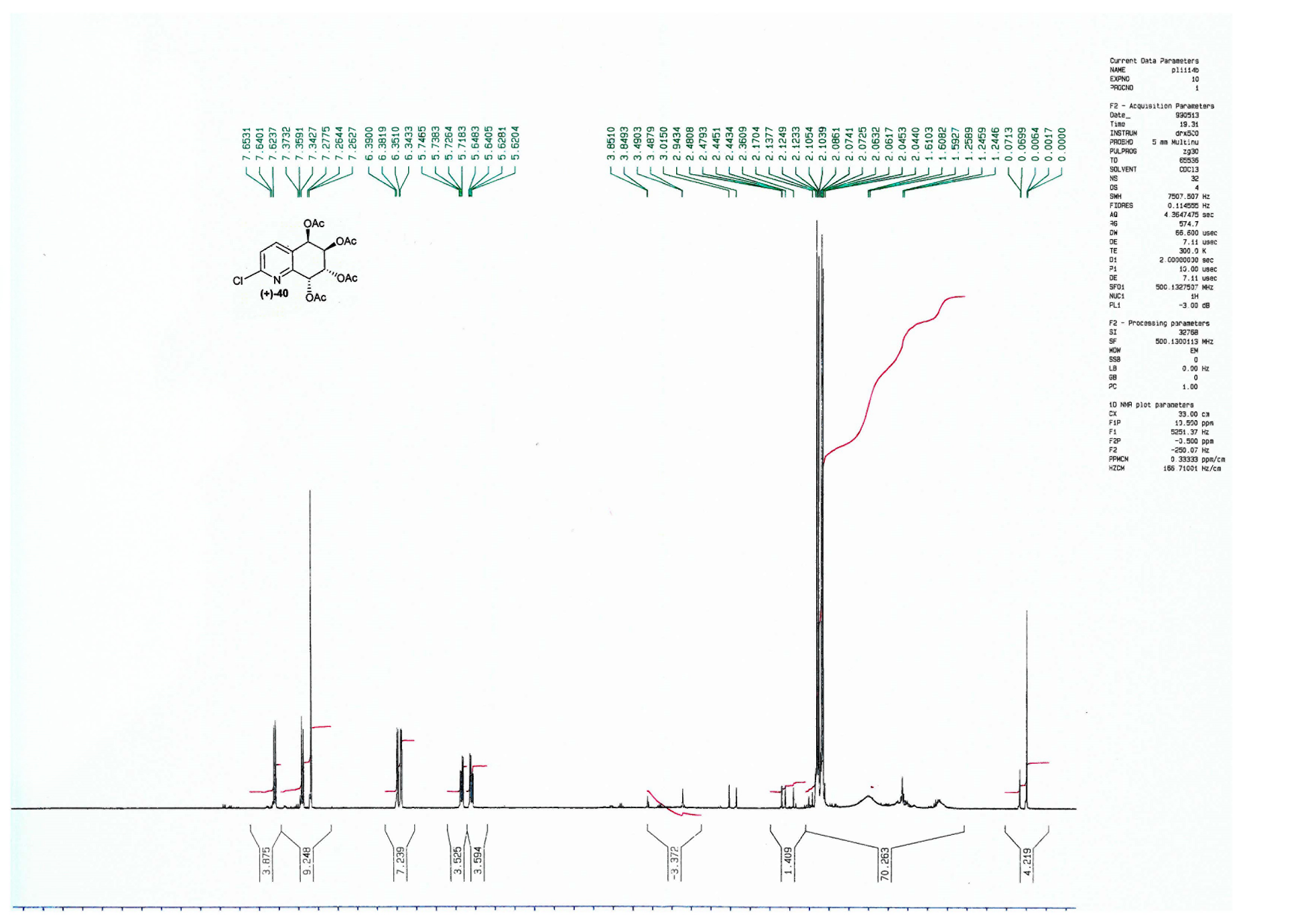

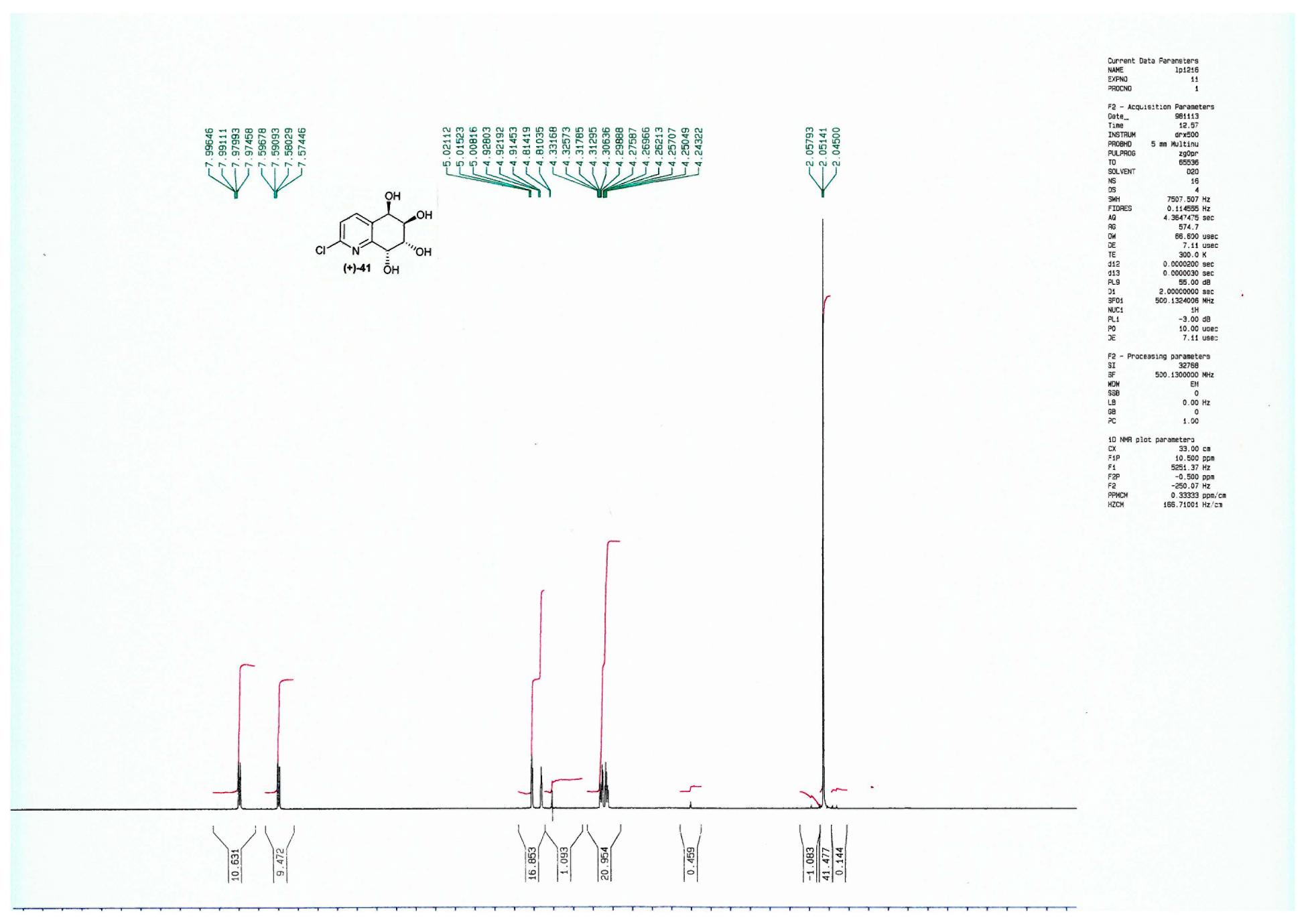
**

**

**

**
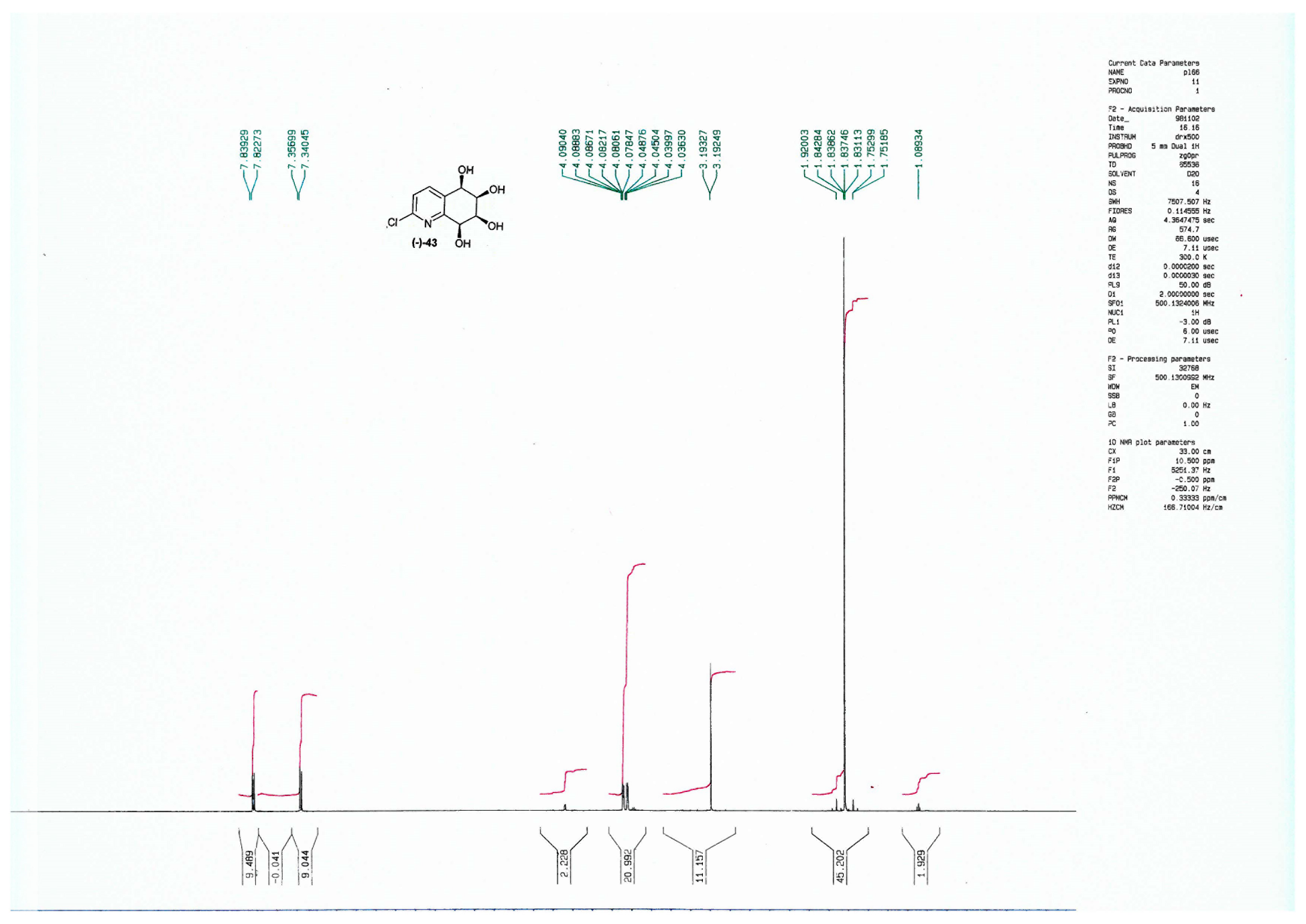

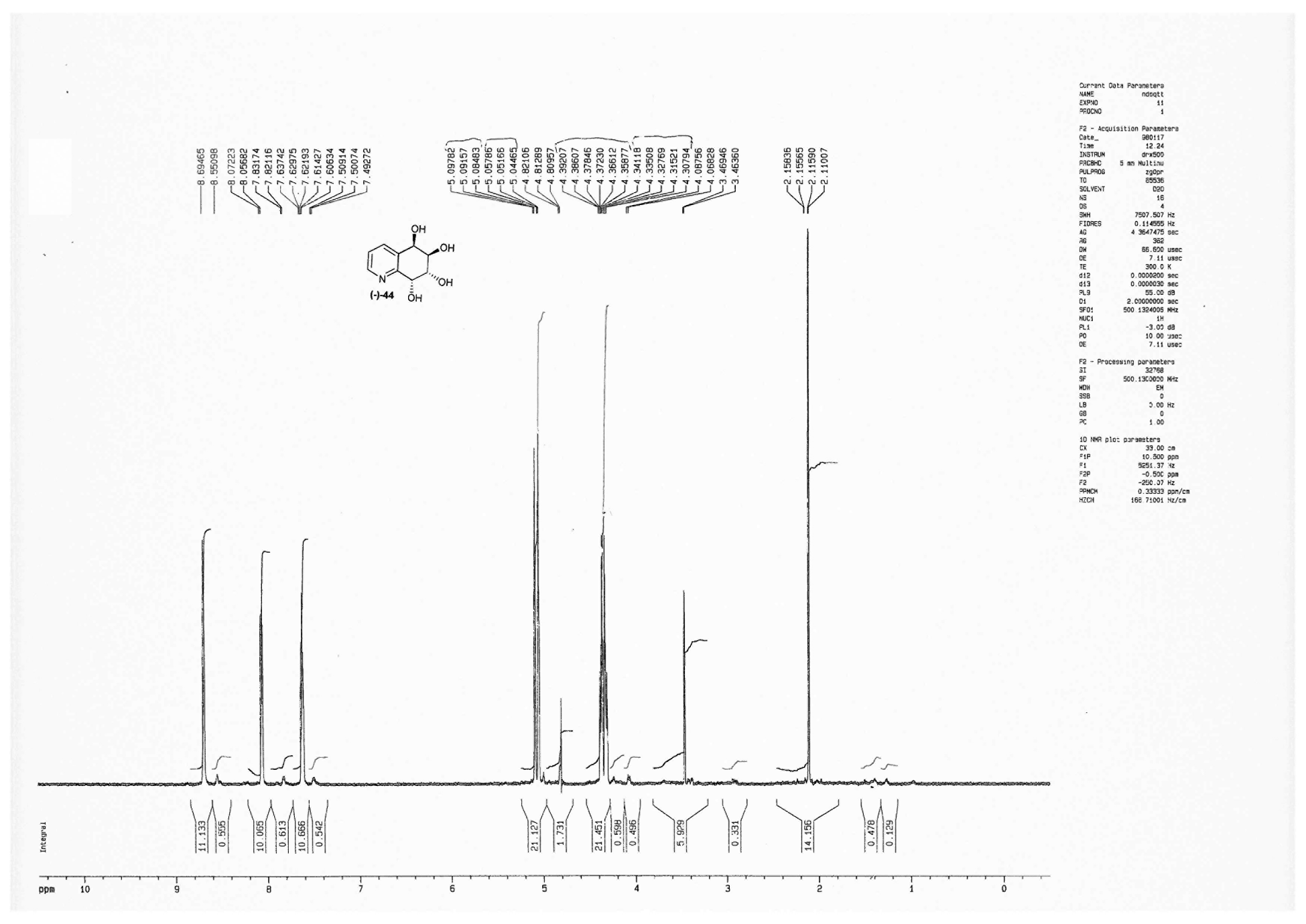

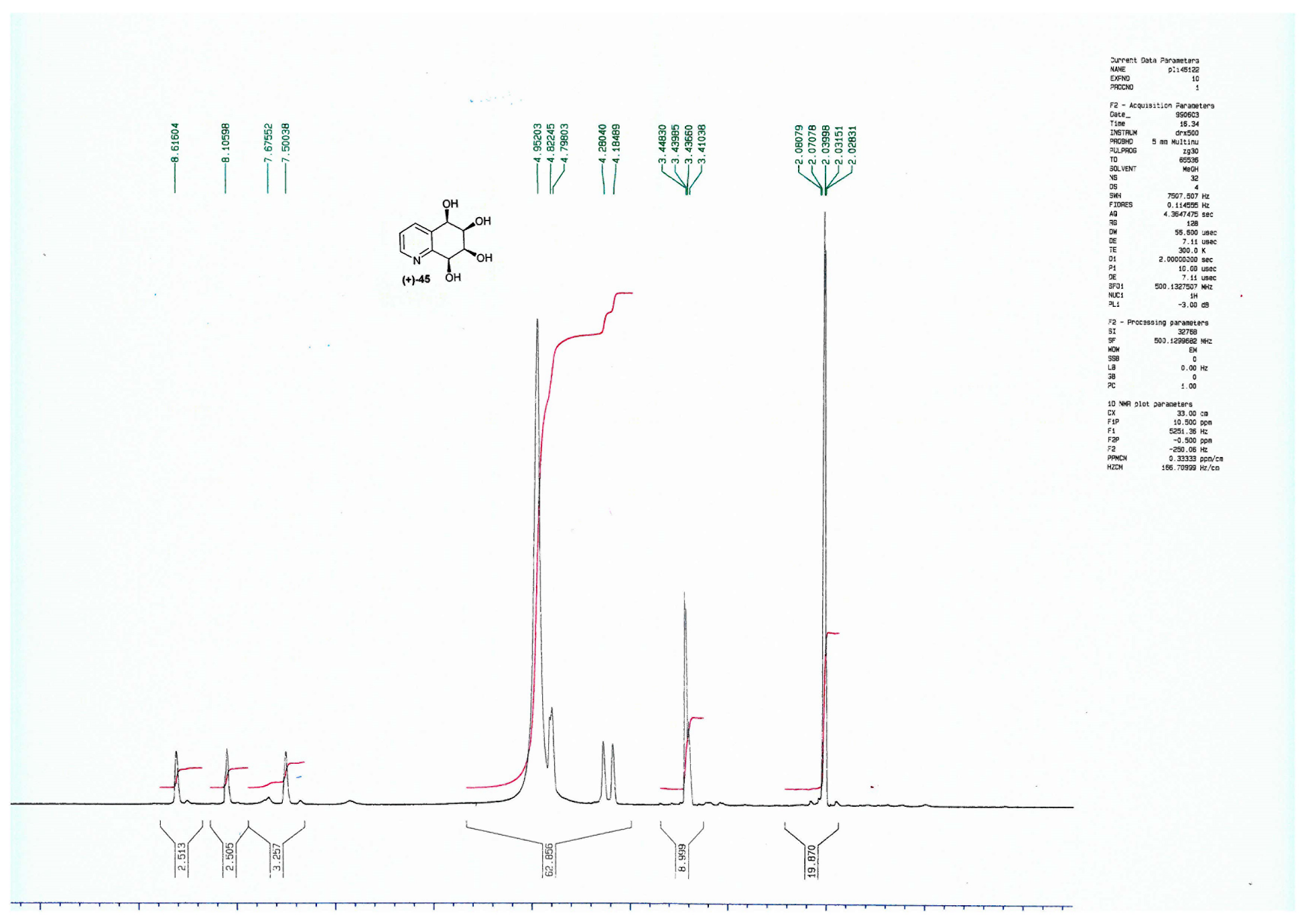

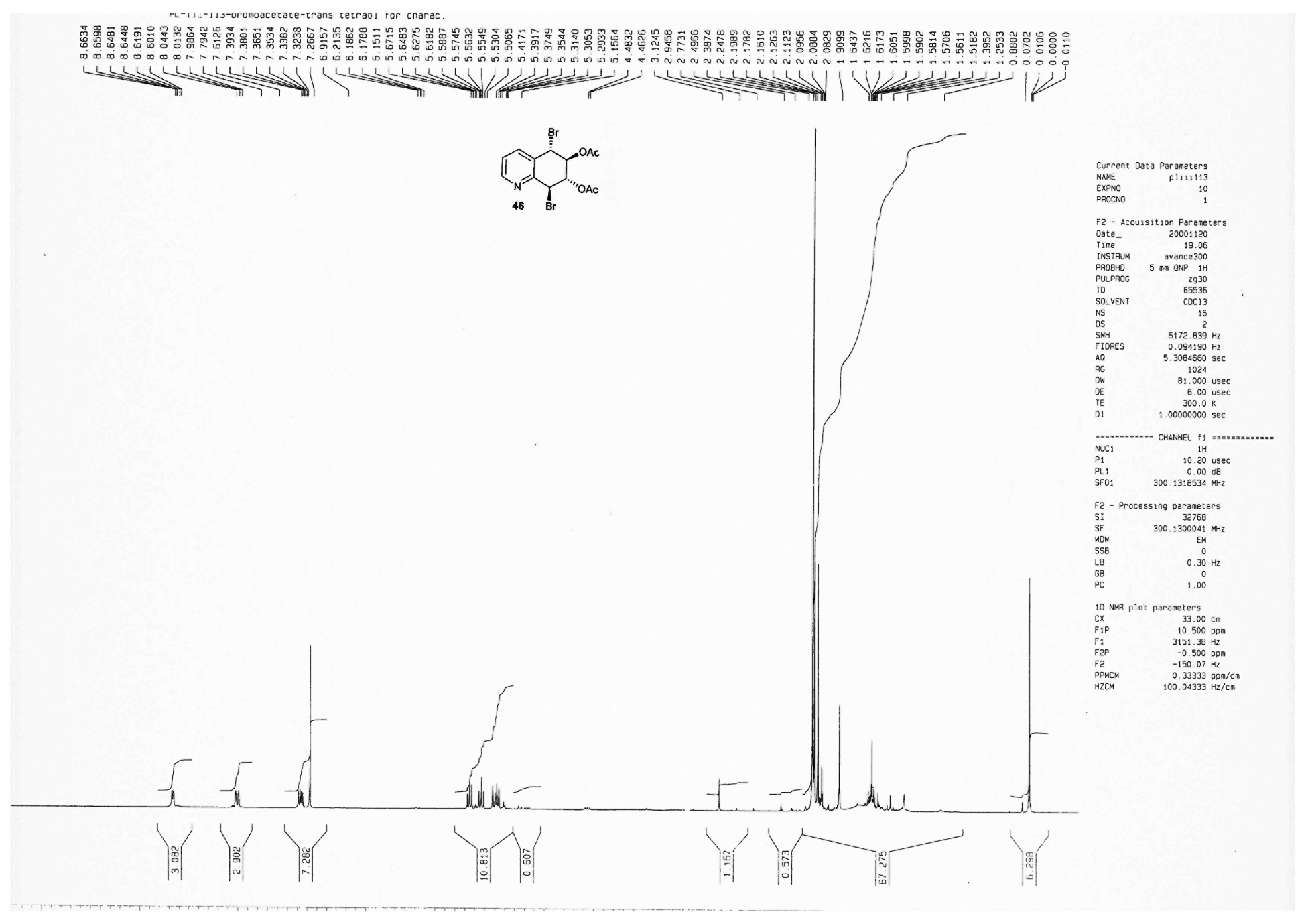

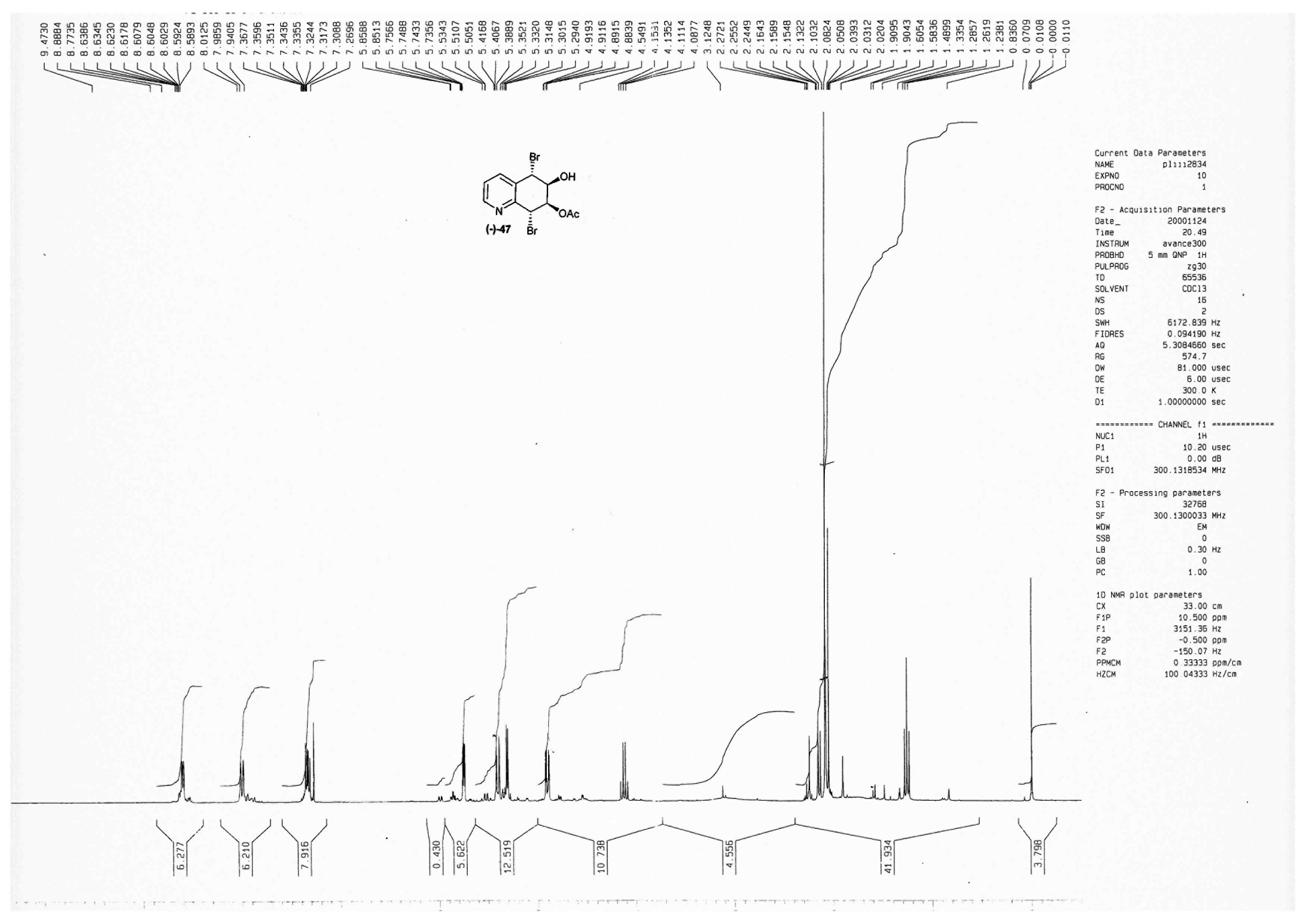
**

**
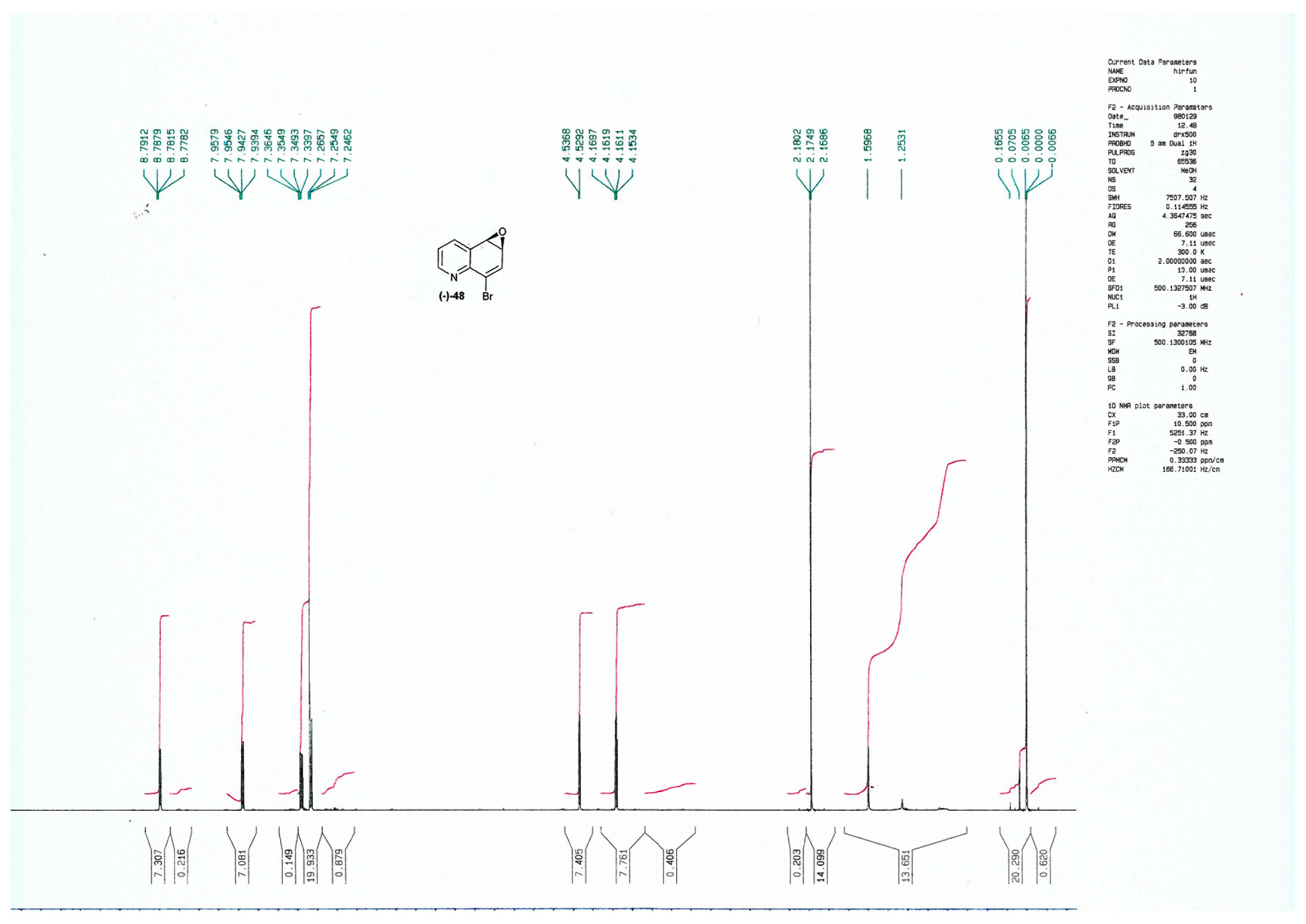
**

**
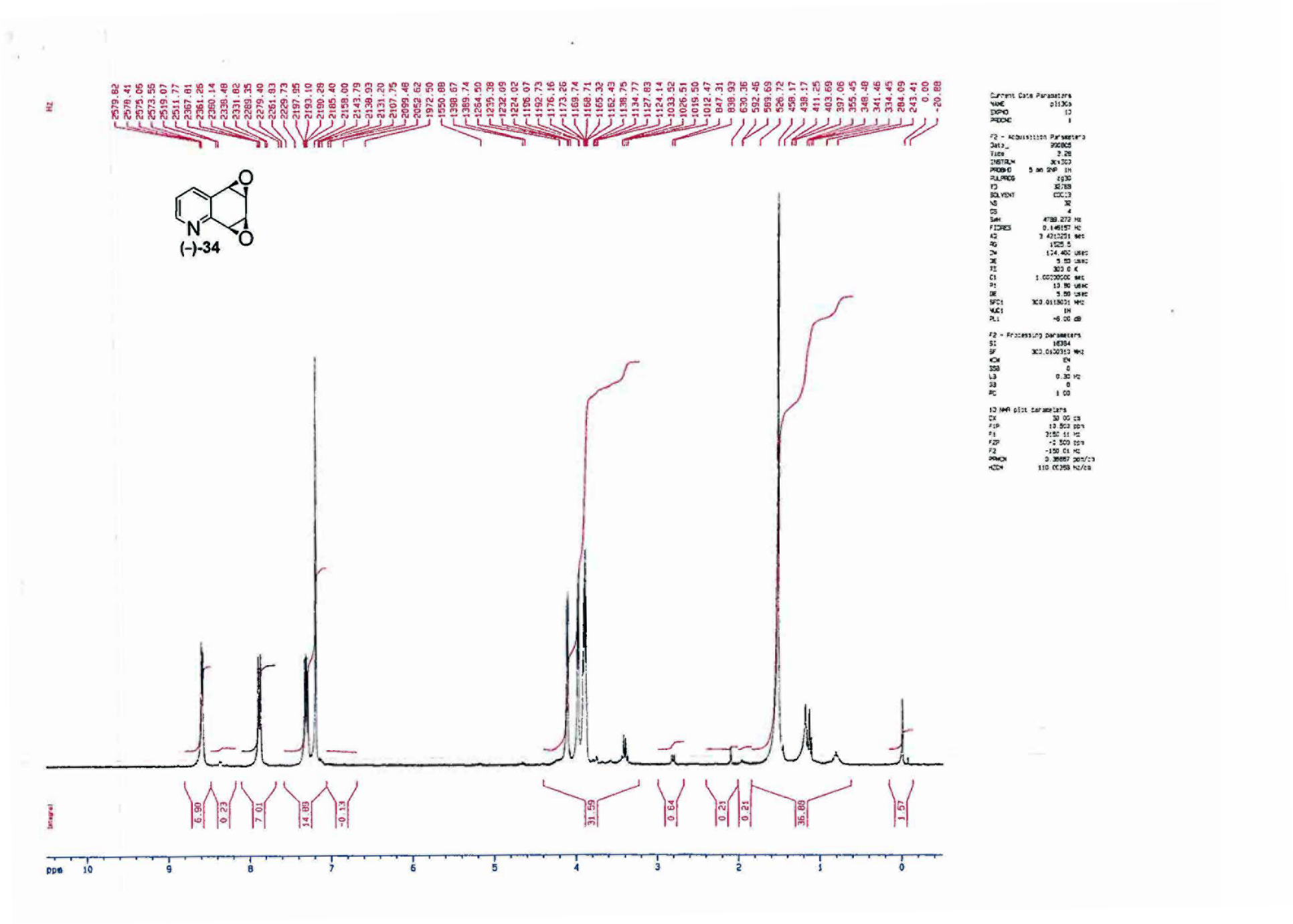
**

**
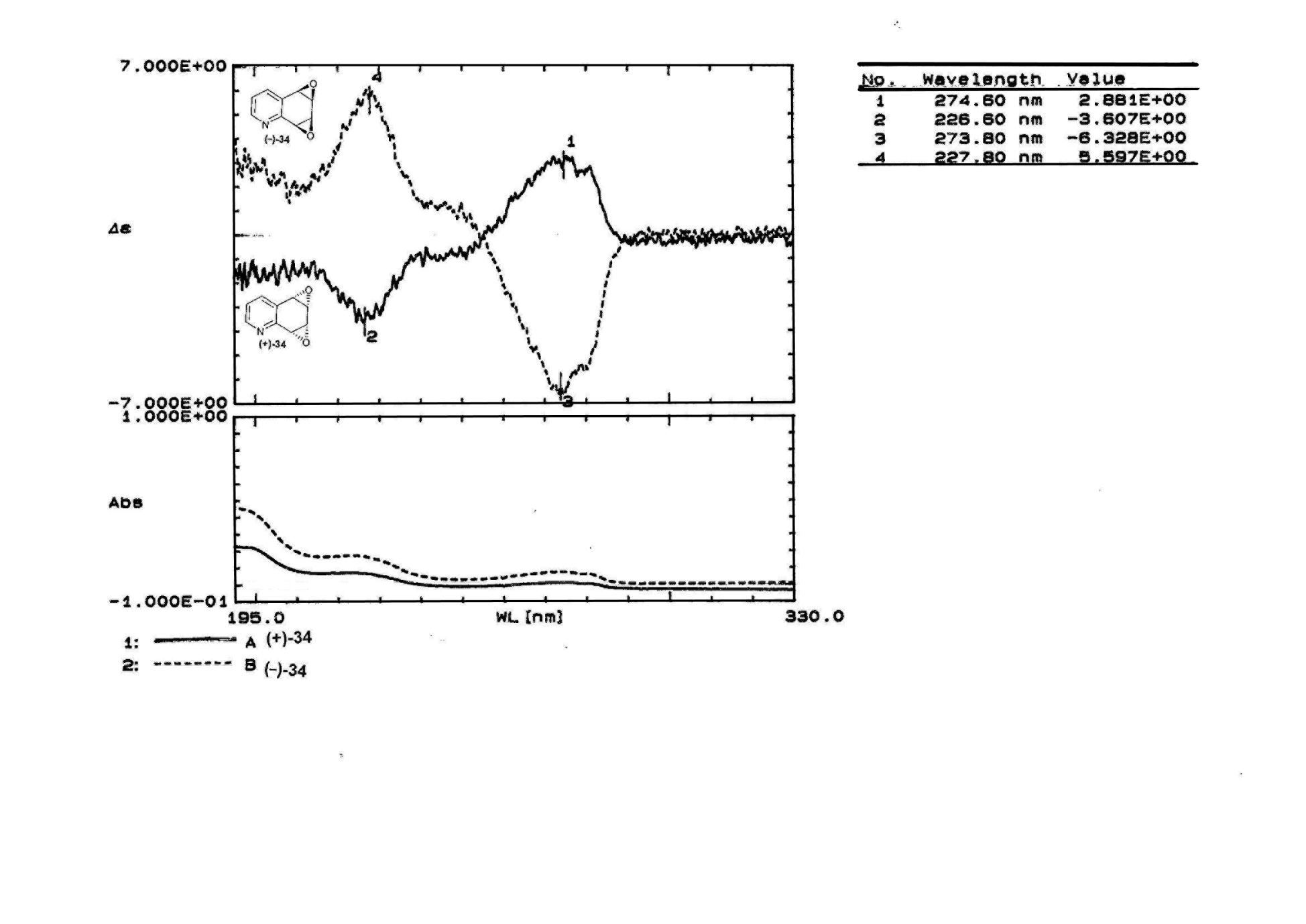
**

His228

His222


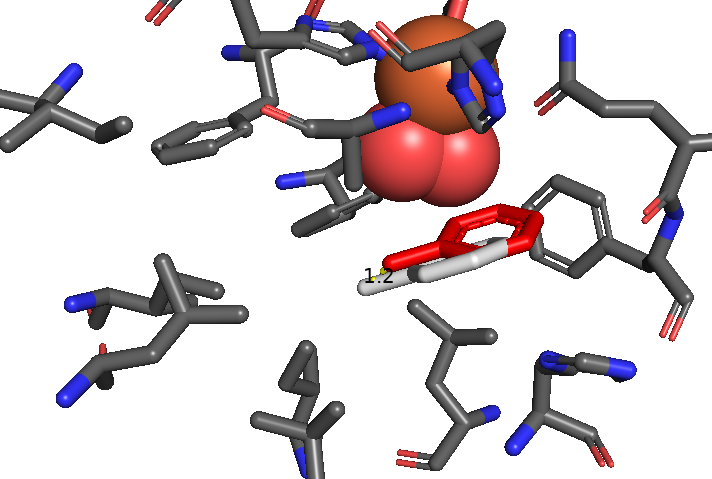


Ile276

Leu272

Ile232

Phe372

Phe366

Ala223

Ile324

Leu321

Val309

His311

Asn215

Phe216

**Figure S-13A: First orientation of docked toluene (grey) with a calculated binding energy of -5.1 kcal/mol compared to toluene as found in the crystal structure (red).**


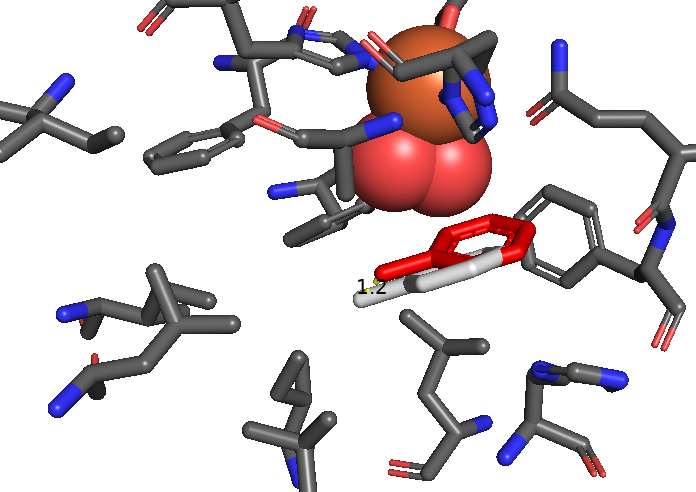


Ile276

Leu272

Ile232

Ala223

Ile324

Leu321

Phe372

Phe366

His228

Val309

His311

His222

Asn215

Phe216

**Figure S-13B: Second orientation of docked toluene (grey) with a calculated binding energy of -5.1 kcal/mol compared to toluene as found in the crystal structure (red).**


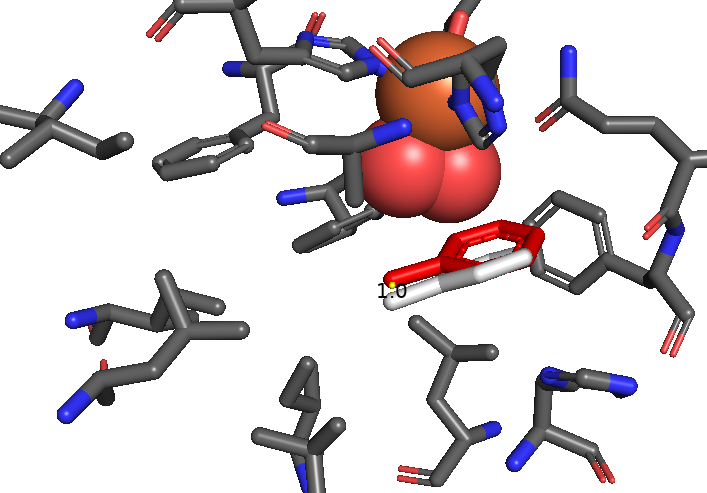


Ile276

Leu272

Ile232

Ala223

Ile324

Leu321

Phe372

Phe366

His228

Val309

His311

His222

Asn215

Phe216

**Figure S-13C: Third orientation of docked toluene (grey) with a calculated binding energy of -4.9 kcal/mol compared to toluene as found in the crystal structure (red).**


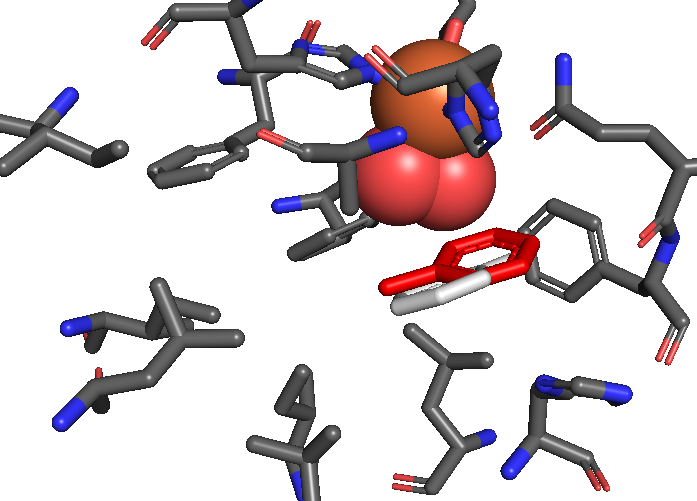


Ile276

Leu272

Ile232

Ala223

Ile324

Leu321

Phe372

Phe366

His228

Val309

His311

His222

Asn215

Phe216

**Figure S-13D: Orientation of docked toluene (grey) with a calculated binding energy of -5.3 kcal/mol compared to toluene as found in the crystal structure (red). The methyl group of this orientation is directed at the Phe216 residue, distancing the aromatic ring from the dioxygen. This orientation is assumed to be of low reactivity, but would result in the opposite enantiomer being formed upon dihydoxylation.**
